# Spatiotemporal subtypes of brain and spinal cord atrophy in neuromyelitis optica spectrum disorders and multiple sclerosis

**DOI:** 10.1186/s12916-025-04366-7

**Published:** 2025-09-02

**Authors:** Zhizheng Zhuo, Xiaolu Xu, Siyao Xu, Shi Yao, Jinyuan Weng, Fuqing Zhou, Tiantian Hua, Jun Sun, Dan Cheng, Guanmei Cao, Xinghu Zhang, Fudong Shi, Tielin Yang, Sven Haller, Andre Altmann, Yuehua Li, Decai Tian, Yunyun Duan, Yaou Liu

**Affiliations:** 1 Department of Radiology, Beijing Tiantan Hospital, Capital Medical University, No.119, the West Southern 4Th Ring Road, Fengtai District, Beijing, 100070 China; 2 National and Local Joint Engineering Research Center of Biodiagnosis and Biotherapy, The Second Affiliated Hospital, Xi’an Jiaotong University, Xi’an, Shaanxi, 710004 P.R. China; 3 Clinical Science, Philips Healthcare, Shanghai, 200235 P.R. China; 4 Department of Radiology, The First Affiliated Hospital, Nanchang University, Nanchang, Jiangxi Province 330006 P.R. China; 5 Center for Neurology, Beijing Tiantan Hospital, Capital Medical University, Beijing, 100070 P.R. China; 6 China National Clinical Research Center for Neurological Diseases, Beijing, 100070 P.R. China; 7 Department of Neurology, Tianjin Neurological Institute, Tianjin Medical University General Hospital, Tianjin, 300052 P.R. China; 8 Key Laboratory of Biomedical Information Engineering of Ministry of Education, Biomedical Informatics & Genomics Center, School of Life Science and Technology, Xi’an Jiaotong University, Xi’an, Shaanxi, 710049 P.R. China; 9 Department of Imaging and Medical Informatics, University Hospitals of Geneva and Faculty of Medicine of the University of Geneva, Geneva, Switzerland; 10 Centre for Medical Image Computing Department of Medical Physics and Biomedical Engineering and Department of Computer Science, University College London, London, UK; 11 Department of Radiology, Shanghai Sixth People’s Hospital Affiliated to Shanghai Jiao Tong University School of Medicine, Shanghai, 201306 P.R. China

**Keywords:** Brain and spinal cord atrophy, Neuromyelitis optica spectrum disorders, Multiple sclerosis, Subtype

## Abstract

**Background:**

Neuromyelitis optica spectrum disorders (NMOSD) and multiple sclerosis (MS) are autoimmune demyelinating diseases with overlapping clinical features but distinct patterns of brain and spinal cord atrophy. The precise atrophy subtypes specific to each disease remain elusive. This study aimed to identify shared and distinct atrophy subtypes in NMOSD and MS, using neuroimaging to explore their clinical significance and potential implications for tailored treatment strategies.

**Methods:**

Clinical and MRI data of 278 AQP4 + NMOSD and 391 MS patients were retrospectively and prospectively collected, alongside 1,065 healthy controls. 3D T1-weighted image derived structural measurements were used in a Subtype and Stage Inference model, to identify distinct brain and spinal cord atrophy subtypes of NMOSD and MS. The clinical characteristics of disease atrophy subtypes and clinical associations of atrophy stage were investigated.

**Results:**

The results showed that in NMOSD, three atrophy subtypes were identified: (1) cortical subtype with severe cognitive and physical disability; (2) spinal cord subtype with high number of relapses; and (3) cerebellum subtype with a favorable prognosis. In MS, three atrophy subtypes were identified: (1) cortical subtype featuring severe cognitive decline; (2) spinal cord subtype featuring high number of relapses; and (3) subcortical subtype featuring severe physical disability. Advanced stages in MS spinal cord and subcortical atrophy subtypes were associated with severe physical disability and cognitive decline, while advanced stage in all MS subtypes correlated with disability worsening.

**Conclusions:**

These novel imaging subtypes in NMOSD and MS may help interpret disease heterogeneity, develop stratified management, and assess prognosis.

**Supplementary Information:**

The online version contains supplementary material available at 10.1186/s12916-025-04366-7.

## Background

Neuromyelitis optica spectrum disorders (NMOSD) and multiple sclerosis (MS) are both autoimmune inflammatory demyelinating conditions of the central nervous system, affecting the optic nerve, brain, and spinal cord [1, 2]. Although MS and NMOSD exhibit overlapping clinical features, their immunopathological underpinnings and neurodegenerative mechanisms are distinct [3, 4]. NMOSD is characterized as an astrocytopathy, where autoantibodies are directed against the aquaporin-4, resulting in astrocyte injury [3]. In contrast, MS primarily impacts oligodendrocytes, but no specific autoantigen has been identified for this disease [4]. Both diseases exhibit brain and spinal cord atrophy, which is correlated with various degrees of cognitive decline and physical disability [5, 6]. Understanding the distinct and shared atrophy patterns in NMOSD and MS can provide crucial insights into their differential disease progression and patient management.

Neuroimaging studies have demonstrated distinct brain and spinal cord atrophy patterns in NMOSD and MS [7–10]. MS is characterized by significant cortical atrophy, deep gray matter (e.g., thalamus) atrophy, and cerebellar involvement, which strongly correlate with cognitive decline [7, 9]. In contrast, cortical atrophy is less pronounced in NMOSD, and thalamic volume loss remains controversial [7–11]. However, spinal cord involvement is more prominent in NMOSD, particularly associated with long lesions and more frequent relapses compared to MS [12–14]. Identifying these phenotypic differences is crucial for disease stratification and targeted therapeutic interventions.

Despite the inherent heterogeneity within the disease, specific MRI-based phenotypes have been proposed for MS, identifying various atrophy patterns in gray and white matter (GM and WM) that align with different disease progressions [15–19]. However, spinal cord atrophy has not been as extensively incorporated into these models [19]. At present, there is a scarcity of well-defined MRI phenotypes for NMOSD [19]. Subtype and Stage Inference (SuStaIn) offers a promising computational tool to uncover shared and distinct spatiotemporal atrophy patterns in NMOSD and MS based on neuroimaging data [20, 21]. The disease progression modeled by SuStaIn may reflect the spatiotemporal advancement of atrophy patterns rather than direct clinical progression (e.g., worsening EDSS scores or relapse rates). In addition, these SuStaIn-derived stages may serve as a structural framework to characterize how atrophy evolves across subtypes over time. This study hypothesized that SuStaIn could delineate the atrophy trajectories in both conditions, potentially informing better disease management strategies and therapeutic approaches tailored to their distinct neurodegenerative profiles.

## Methods

### Study design

This study aimed to analyze spatiotemporal patterns of brain and spinal cord atrophy in aquaporin-4 antibody positive (AQP4 +) NMOSD and MS using SuStaIn Model analysis (Additional file 1: Table S1 and Supplementary Results). The first aim was to identify shared and distinct atrophy subtypes between AQP4 + NMOSD and MS. The second aim assessed subtype stability and progression using follow-up MR scans. The third aim characterized baseline clinical features, disability progression, and additional MRI characteristics for each atrophy subtype.

### Participants

The clinical and MRI data of 278 AQP4 + NMOSD (age = 43 [31, 53] years, [median, interquartile range [IQR]]; female ratio = 256/278) and 391 relapsing–remitting MS patients (age = 34 [27, 42] years; female ratio = 264/391) were retrospectively (167 AQP4 + NMOSD and 235 MS) and prospectively (111 AQP4 + NMOSD and 156 MS) collected from a Chinese multicenter neuroinflammatory disease database (Table 1, Additional file 1: Fig. S1-S2 and Supplementary Results). One thousand and sixty-five healthy volunteers in the same database (*n* = 361) and a local lifespan cohort (*n* = 704) were recruited as healthy controls (HCs) (Table 1). A subset of recruited patients had follow-up clinical data on Expanded Disability Status Scale (EDSS) and relapse (176 MS and 89 AQP4 + NMOSD) as well as MR scans (33 MS and 28 AQP4 + NMOSD). NMOSD was diagnosed based on the 2015 International Panel [22]. AQP4 + NMOSD cases were seropositive for AQP4 antibody but negative for myelin oligodendrocyte glycoprotein antibody by cell-based assay [23]. Relapsing–remitting MS cases were diagnosed based on 2017 McDonald criteria [24] and seronegative for both AQP4 and myelin oligodendrocyte glycoprotein antibodies. Other inclusion criteria were: (1) MRI performed four or more weeks from the last attack (to exclude the effect of the acute phase on MRI measurements); (2) age between 16 and 65 years. Exclusion criteria were: (1) contradiction to MRI; (2) incomplete MRI acquisition or poor image quality; (3) a history of other neurological or neuropsychological diseases (e.g., stroke or dementia).

**Table 1:** Clinical and MRI characteristics of atrophy subtypes in AQP4 + NMOSD and MS

	HC (*n* = 1065)	NMOSD-NA (*n* = 107)	NMOSD-C (*n* = 87)	NMOSD-SC (*n* = 58)	NMOSD-CE (*n* = 26)	p-NMOSD	MS-NA (*n* = 123)	MS-C (*n* = 72)	MS-SC (*n* = 115)	MS-DGM (*n* = 81)	p-MS
**Demographics**											
Age, years	45.0 [31.0, 54.0]	43.0 [30.5, 52.0]	43.5 [28.0, 51.0]	43.0 [32.5, 57.0]	40.5 [31.5, 50.5]	0.39	34.5 [26.0, 43.0]	33.0 [26.5, 40.5]	36.0 [25.5, 44.0]	34.0 [30.5, 41.0]	0.73
Female, number (percentage)	566 (53.15%)	101 (94.4%)	82 (94.3%)	52 (89.7%)	21 (80.8%)		82 (66.7%)	48 (66.7%)	75 (65.8%)	59 (72.8%)	
Education (years, number)	16.0 [13.0, 16.0] (886)	12.0 [9.0, 16.0] (64)	12.0 [9.0, 16.0] (62)	12.0 [9.0, 13.5] (37)	12.0 [9.0, 16.0] (22)	0.98	16.0 [13.5, 16.0] (89)	12.0 [9.0, 16.0] (52)	15.0 [9.5, 16.0] (84)	16.0 [9.0, 16.0] (66)	0.084
**Clinical information**											
Disease duration (months)	NA	22.4 [6.9, 57.3] (94)	25.6 [6.6, 94.6] (76)	44.0 [12.0, 62.0] (47)	17.6 [8.5, 42.8] (24)	0.29	17.7 [7.0, 45.0] (105)	28.0 [7.1, 71.0] (58)	30.8 [12.0, 84.0] (104)	36.0 [15.0, 82.5] (71)	0.006
Baseline number of relapses	NA	2.0 [1.0, 3.0] (50)	2.0 [1.0, 5.0] (59)	3.0 [2.0, 4.0] (27)	2.0 [1.0, 3.0] (18)	0.014	2.0 [1.0, 3.0] (51)	2.0 [1.0, 3.0] (37)	3.0 [2.0, 5.0] (49)	2.0 [2.5, 3.5] (47)	0.002
Bassline EDSS	NA	3.0 [1.0, 4.0] (74)	3.5 [2.0, 5.5] (67)	3.5 [1.5, 5.5] (38)	3.5 [3.0, 4.5] (20)	0.0080	1.5 [1.0, 2.5] (107)	2.0 [1.0, 3.5] (58)	2.5 [1.5, 3.5] (107)	2.0 [1.5, 3.5] (73)	< 0.001
CVLT	85.0 [53.0, 100.0] (289)	84.5 [60.0, 100.0] (64)	87.0 [60.5, 100.0] (61)	76.0 [55.5, 99.0] (37)	98.0 [72.5, 104.0] (20)	0.43	89.0 [67.5, 99.5] (82)	75.0 [65.0, 95.0] (51)	67.0 [47.5, 98.5] (84)	70.0 [61.0, 89.0] (55)	0.16
BVLT	31.0 [26.0, 39.5] (189)	40.0 [24.5, 50.5] (64)	39.0 [27.0, 47.0] (61)	30.0 [19.0, 38.0] (37)	37.0 [23.0, 46.0] (20)	0.011	48.0 [29.5, 54.5] (82)	43.5 [32.0, 48.0] (50)	34.0 [27.0, 51.0] (83)	33.0 [25.0, 46.0] (55)	0.22
PASAT	52.0 [43.5, 57.0] (208)	44.0 [35.0, 53.0] (64)	41.0 [30.5, 49.0] (61)	45.0 [30.0, 57.0] (37)	41.5 [30.5, 44.5] (20)	0.47	54.5 [48.5, 89.5] (82)	47.0 [38.5, 52.5] (51)	42.0 [34.0, 49.5] (84)	45.0 [34.5, 50.0] (55)	< 0.001
SDMT	52.0 [35.5, 60.0] (187)	41.0 [33.0, 50.5] (46)	41.0 [32.0, 51.0] (39)	48.5 [30.5, 57.5] (25)	42.0 [31.5, 49.0] (18)	0.86	55.5 [50.0, 57.5] (61)	42.0 [34.0, 47.0] (35)	50.0 [44.0, 55.0] (44)	47.0 [35.0, 54.0] (29)	0.023
COWAT	26.5 [24.5, 29.5] (187)	18.0 [12.5, 23.0] (46)	12.0 [9.0, 15.5] (39)	20.0 [14.0, 23.0] (25)	22.0 [17.5, 25.5] (18)	0.0010	15.5 [13.5, 18.5] (61)	12.0 [10.5, 17.5] (35)	17.0 [13.0, 19.5] (44)	13.0 [10.0, 16.0] (29)	0.075
Follow-up time (months)	NA	38.5 [32.5, 43.2] (25)	38.4 [25.8, 44.2] (38)	40.2 [32.4, 61.8] (17)	33.6 [33.6, 42.0] (9)	0.66	40.8 [33.6, 70.0] (37)	42.0 [32.4, 92.0] (33)	61.0 [42.0, 70.0] (68)	41.0 [33.0, 61.0] (38)	0.035
Follow-up number of relapses	NA	1.0 [0.0, 1.0] (25)	0.0 [0.0, 1.0] (38)	0.0 [0.0, 1.5] (17)	0.0 [0.0, 1.0] (9)	0.88	0.0 [0.0, 1.0] (37)	0.0 [0.0, 1.0] (33)	1.0 [0.0, 2.0] (68)	0.0 [0.0, 1.00] (38)	0.18
Follow-up EDSS	NA	3.0 [2.0, 4.0] (25)	3.5 [2.0, 4.5] (38)	3.0 [1.5, 4.0] (17)	2.0 [1.5, 3.5] (9)	0.83	1.5 [0.0, 2.5] (37)	3.0 [1.0, 4.5] (33)	3.0 [1.5, 5.5] (68)	3.0 [2.0, 4.0] (38)	0.0020
**Treatment information**											
Disease-modifying therapy, number	NA	44	44	31	11	0.35	59	55	101	57	< 0.001
Immunosuppressant, number	NA	63	43	27	15		64	17	13	24	
**MRI feature**											
WMH volume (ml)	NA	3.5 [0.02, 23.2] (64)	2.7 [0.1, 15.7] (63)	2.8 [0.2, 45.4] (29)	1.0 [0.2, 9.3] (8)	0.14	8.0. [0.1, 18.8]	9.2 [0.5, 16.4]	10.2 [0.8, 38.1]	12.4 [1.0, 52.5]	0.080
Choroid plexus volume (ml)	1.84 [1.48, 2.21] (1065)	1.69 [1.46, 1.93] (107)	1.87 [1.55, 2.23] (87)	1.87 [1.53, 2.17] (58)	1.82 [1.56, 2.11] (26)	0.022	1.96 [1.61, 2.35] (123)	2.10 [1.69, 2.70] (72)	2.28 [1.89, 2.57] (114)	2.16 [1.77, 2.57] (81)	< 0.001
Cerebral WM FA	0.36 [0.34, 0.38] (903)	0.36 [0.35, 0.38] (80)	0.35 [0.33, 0.36] (69)	0.35 [0.34, 0.36] (43)	0.35 [0.34, 0.36] (24)	0.029	0.35 [0.34, 0.37] (102)	0.35 [0.32, 0.36] (65)	0.33 [0.31, 0.35] (93)	0.33 [0.32, 0.35] (72)	< 0.001
Cerebellar WM FA	0.32 [0.29, 0.36] (903)	0.35 [0.33, 0.37] (80)	0.36 [0.33, 0.37] (69)	0.35 [0.31, 0.37] (43)	0.35 [0.34, 0.36] (24)	0.928	0.34 [0.31, 0.37] (102)	0.33 [0.31, 0.36] (65)	0.30 [0.25, 0.35] (93)	0.33 [0.29, 0.34] (72)	< 0.001
Brainstem FA	0.32 [0.29, 0.34] (903)	0.27 [0.25, 0.30] (80)	0.26 [0.24, 0.27] (69)	0.26 [0.24, 0.31] (43)	0.25 [0.25, 0.25] (24)	0.026	0.27 [0.25, 0.31] (102)	0.27 [0.24, 0.29] (65)	0.26 [0.24, 0.29] (93)	0.27 [0.23, 0.30] (72)	0.0094
Cerebral GM fALFF	0.13 [0.11, 0.16] (903)	0.12 [0.09, 0.14] (79)	0.12 [0.08, 0.13] (65)	0.12 [0.10, 0.14] (43)	0.12 [0.05, 0.14] (23)	0.85	0.12 [0.11, 0.15] (100)	0.13 [0.09, 0.15] (65)	0.13 [0.10, 0.15] (91)	0.14 [0.11, 0.17] (72)	0.30
Cerebellar GM fALFF	−0.29 [−0.46, −0.18]	−0.21 [−0.28, −0.12] (79)	−0.18 [−0.33, −0.03] (65)	−0.24 [−0.35, −0.15] (43)	−0.14 [−0.22, −0.02] (23)	0.025	−0.26 [−0.36, −0.15] (100)	−0.24 [−0.37, −0.13] (65)	−0.26 [−0.38, −0.16] (91)	−0.33 [−0.55, −0.19] (72)	0.20

Categorical data were presented by percentage and compared using Pearson’s Chi-squared test. Continuous and ranked data were presented by median and interquartile range and compared by Kruskal–Wallis test between atrophy subtypes. The available case numbers are given in parentheses where relevant. Disease-modifying therapies included teriflunomide, fingolimod and Siponimod. Immunosuppressants, including rituximab, tocilizumab, cyclophosphamide and azathioprine

*CVLT* California Verbal Learning Test, *BVMT* Brief Visuospatial Memory Test, *PASAT* Paced Auditory Serial Addition Test, *SDMT* Symbol Digit Modalities Test, *COWAT* Controlled Oral Word Association Test, *EDSS* Expanded Disability Status Scale, *WMH* White matter hyperintensity, *GM* Gray matter, *FA* Fractional anisotropy, *fALFF* Fractional amplitude of low-frequency fluctuation, *HC* Healthy control, *NMOSD* Neuromyelitis optica spectrum disorders, *MS* Multiple sclerosis, *NMOSD-NA* “normal-appearing” AQP4 antibody positive (AQP4 +) NMOSD, *NMOSD-C* Cortical atrophy leading subtype of AQP4 + NMOSD, *NMOSD-SC* Spinal cord atrophy leading subtype of AQP4 + NMOSD, *NMOSD-CE* Cerebellar atrophy leading subtype of AQP4 + NMOSD, *MS-NA* “normal-appearing” MS, *MS-C* Cortical atrophy leading subtype of MS, *MS-SC* Spinal cord atrophy subtype of MS, *MS-DGM* Deep gray matter atrophy subtype of MS

This study was approved by the institutional review board of Beijing Tiantan Hospital, Capital Medical University, Beijing, China (No. KY 2019–050-02). Written informed consent was obtained from each participant according to the Declaration of Helsinki. The use of external datasets was approved by local ethics committees.

### Clinical and cognitive measures

Clinical variables at baseline and follow-up (available in a subset of patients) were recorded, including age, sex, education, disease duration, number of relapses, EDSS score and treatment information (Table 1). Clinical disability progression was defined as an EDSS score increase ≥ 1.0 for baseline EDSS ≤ 5.5 or an EDSS score increase ≥ 0.5 for baseline EDSS > 5.5. Conversion from relapsing–remitting MS to secondary progressive MS (SPMS) was defined as disability progression occurring over a period of at least 6 months in the absence of a relapse and a resulting EDSS score of 4 or more [25].

Cognitive tests included California Verbal Learning Test, Third Edition (CVLT-III), Brief Visuospatial Memory Test-Revised (BVMT-R) and Paced Auditory Serial Addition Test (PASAT, 3 s), Symbol Digit Modalities Test (SDMT), and Controlled Oral Word Association Test (COWAT). Details on these cognitive tests are provided in Additional file 1: Supplementary Methods.

### MRI acquisition

MR imaging sequences, including 3D T1-weighted (T1W) imaging, fluid-attenuated inversion recovery (FLAIR), diffusion tensor imaging and resting-state functional MR imaging, were performed using 3.0 Tesla MR scanners. Details of MR acquisition protocols are provided in Additional file 1: Table S1.

### Image analysis and post-processing

All MR images were reviewed by an experienced radiologist (X.X., eight years in radiology) for quality control regarding artifacts, contrast, and signal-to-noise ratio. We segmented brain tissue (total intracranial volume [TIV], cerebral WM and GM, cerebellar WM and GM, and brainstem) using white matter hyperintensity (WMH) filled 3D T1W images with the “recon-all” pipeline in FreeSurfer (version 7.3.2). Regional GM volumes (frontal, temporal, parietal, occipital, insula, cingulate, thalamus, hippocampus, caudate, putamen, pallidum, amygdala, nucleus accumbens, and ventral diencephalon) were extracted from “aparc.volume” and “aseg.vol” files. Mean upper cervical cord areas (MUCCAs) at C1 to C3 levels were determined using 3D T1W images with Spinal Cord Toolbox (version 6.3). The 3D T1W sagittal images were acquired for comprehensive coverage of the whole brain and extended to include the cervical spinal cord, at least from the C1 to C3 vertebrae. For the above global and regional brain tissue volumes and MUCCAs, we applied “removeBatchEffect” in the “limma” package to eliminate potential batch effects from scanner protocols and adjust for age, sex, and TIV while preserving group effects (HC, AQP4 + NMOSD, and MS). Z-scores for the adjusted global and regional brain tissue volumes and MUCCAs were calculated relative to age- and sex-matched HCs (R package “MatchIt” with a ratio of 1:1) using generalized linear models, serving as inputs for the SuStaIn model.

We segmented WMH and calculated their volumes using a custom WMH segmentation pipeline on FLAIR images [26], with verification and adjustments by Y.D., a radiologist with 14 years of neuroradiology experience, using ITK-SNAP (Version 3.8.0). Choroid plexus volume, important for assessing neuroinflammation [27], was extracted using a deep learning segmentation algorithm on T1W images [28]. Fractional anisotropy (FA) from diffusion tensor imaging characterizing the microstructural integrity of cerebral and cerebellar WM and the brainstem, and fractional amplitude of low-frequency fluctuation (fALFF) from resting-state functional MR images assessing functional activity in cerebral and cerebellar GM were extracted [29]. The “removeBatchEffect” function in the “limma” package was employed to eliminate potential batch effects from acquisition protocols and adjust for age and sex (and TIV for choroid plexus volume), while preserving group effects (HC, AQP4 + NMOSD, and MS) before statistical analyses. Further details on image processing are provided in the Additional file 1: Supplementary Methods.

### Subtype and stage inference

SuStaIn was used to identify subgroups of AQP4 + NMOSD and MS based on distinct brain and spinal cord atrophy profiles from cross-sectional imaging data. It simultaneously clusters individuals into spatial subtypes and reconstructs temporal atrophy trajectories (set of stages) using disease progression modeling [20]. Each atrophy pattern is defined by a piecewise linear Z-score model, with multiple stages corresponding to biomarkers (e.g., brain region volume or MUCCA adjusted for age, sex, TIV, and scanner) reaching Z-scores of 1, 2, and 3. Stages represent the progression of imaging biomarkers from normal to abnormal. This framework allows the identification of shared spatiotemporal patterns of brain and spinal cord atrophy across populations with different diseases. The optimal number of subtypes was determined via tenfold cross-validation (Additional file 1: Fig. S3, cross-validation information criterion [CVIC]) to balance model complexity and accuracy [20]. The model with the lowest CVIC was selected, prioritizing lower complexity when CVICs were similar. In this study, gradual atrophy refers to the stepwise progression of atrophy biomarkers (e.g., brain or spinal cord volumes) across SuStaIn stages, reflecting a temporal trajectory of atrophy within a given subtype. By contrast, atrophy generally refers to the loss of tissue volume without indicating progression over time.

### Assigning individuals to subtypes and stages

Individuals were subtyped by comparing their likelihood of belonging to each SuStaIn subtype (summing over SuStaIn stage) with the likelihood of being at SuStaIn stage 0 (e.g., with no imaging abnormalities). Cases with a higher probability of belonging to SuStaIn stage 0 than any other SuStaIn subtype were termed “normal-appearing” (NA, findings on “normal-appearing” patients were found in Additional file 1: Fig. S4-S5). Individuals with a higher probability of belonging to a SuStaIn subtype than stage 0 were considered to be “subtypeable”. Each subtypeable individual was then assigned to their most probable subtype. Individuals were staged by computing their average SuStaIn stage, weighted by their probability of belonging to each stage of each subtype.

### Statistical analyses

Statistical analysis was performed using R software (version 4.1.3). Categorical data were presented as percentages and analyzed with the Pearson’s Chi-squared test. Continuous and ranked data were expressed as median and interquartile range (IQR) and compared using the Kruskal–Wallis test, followed by post-hoc analysis with false discovery rate (FDR) correction.

The optimal SuStaIn model derived from baseline data was subsequently applied to a subset (*n* = 61) of available longitudinal MRI scans. Descriptive statistics were used to determine subtype stability (proportion of subjects with the same subtype at follow-up) and stage progression (proportion of subjects with lower, same or higher stage at follow-up).

Subtypes were statistically compared to one another and to NA (that is, stage 0) individuals to determine subtype-specific features. Overall differences between subtypes were assessed independently of stage after adjustment for age, sex and disease duration (except when the variable itself was evaluated, and education level was additionally adjusted for cognitive scores) by the Kruskal–Wallis test followed by post-hoc comparison both without and with FDR correction between groups because of the exploratory nature of this study (details in Additional file 1: Supplementary Results). Sex and treatment differences between subtypes were tested by the Pearson’s Chi-squared test. These analyses compared age, sex, education level, disease duration, number of relapses, EDSS, cognitive scores, and treatment. Additional structural and functional MR indexes including WMH volume, choroid plexus volume, FA and fALFF were also compared. One-versus-all comparisons between subtypes were also conducted to examine the features of each subtype.

Spearman’s correlation analysis was performed to assess the relationships between SuStaIn stage and clinical variables (age, disease duration, number of relapses, EDSS, cognitive scores), lesion- and inflammation-related indexes (WMH volume and choroid plexus volume). In these analyses, stage was correlated with these variables after adjustment for age, sex and disease duration (except when the variable itself was evaluated), and the reported results were stratified by subtype. Additional adjusting education level for cognitive scores was performed. Stage analyses in AQP4 + NMOSD and MS without stratification by subtype are provided in Additional file 1: Supplementary Results.

Both univariate and multivariate Cox proportional-hazards regression models (additional step-wise backward regression was conducted using “autoReg” package in R) were utilized to investigate potential risk factors, including atrophy subtypes, stage, age, sex and disease duration, for follow-up disability worsening and relapse.

The additional treatment responses to disease-modifying therapies (DMT) were evaluated among patients with AQP4 + NMOSD and MS subtypes. Clinical evidence has established the efficacy of DMT in these patient populations; however, treatment responses have been observed to be highly variable. Given the absence of follow-up FLAIR imaging in our cross-sectional dataset, we are unable to ascertain the lesion response to DMT. Consequently, we relied solely on subsequent relapses and EDSS scores to ascertain whether patients experienced relapses or worsening of EDSS during clinical follow-ups, thereby assessing the treatment response to DMT. For relapse, a responder was defined as a patient with no relapse during follow-up. For EDSS, a responder was defined as a patient with no EDSS worsening during follow-up. The Fisher’s exact test was performed to assess the difference in response rate for AQP4 + NMOSD and MS, respectively. All statistical comparisons were conducted in a two-tailed hypothesis manner with FDR correction when available.

## Results

### Demographic data

Totally 1,774 subjects were initially enrolled, including 1,082 HCs, 290 AQP4 antibody positive neuromyelitis optica spectrum disorders (AQP4 + NMOSD) and 402 multiple sclerosis (MS) cases. Fourteen HCs, 6 AQP4 + NMOSD and 6 MS were excluded due to a history of other CNS disease. Three HCs, 6 AQP4 + NMOSD and 5 MS were excluded due to poor image quality. Finally, 1,734 subjects, including 1,065 HCs (age = 45 [31, 54], median [interquartile range, IQR] female percentage = 566/1,065), 278 AQP4 + NMOSD (age = 43 [31, 53] years; female percentage = 256/278) and 391 MS (age = 34 [27, 42] years, female percentage = 264/391) were included in this study (Table 1).

### Brain and spinal cord spatiotemporal atrophy subtypes in AQP4 + NMOSD and MS

**Fig. 1:**
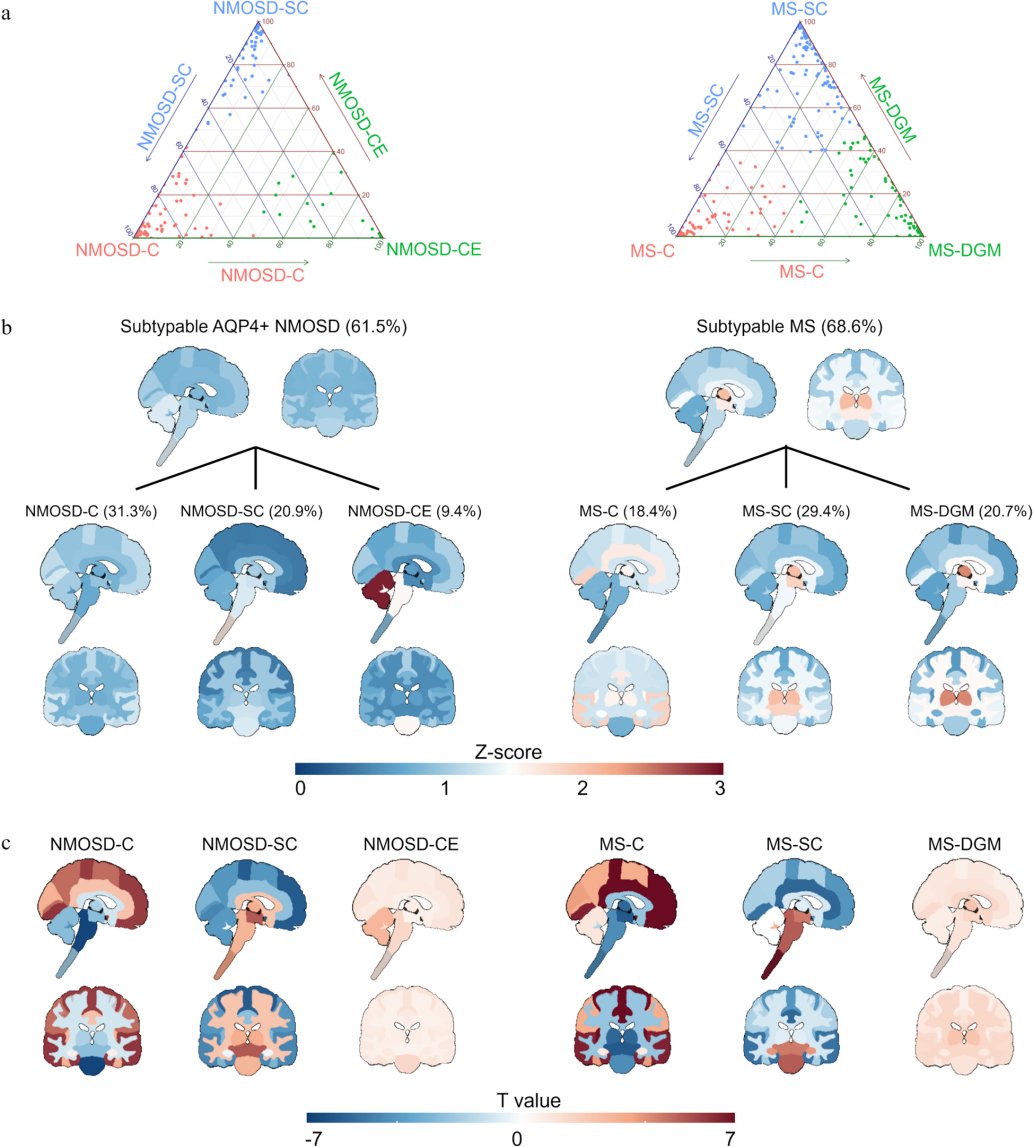
SuStaIn subtypes of AQP4 + NMOSD and MS. **a**, Ternary plot showing the probability of each individual to be classified in a subtype. Dots are labeled by final subtype classification. **b**, Averaged z-score mappings of brain and spinal cord volumes for each disease and its subtypes. The positive Z-score, indicating atrophy compared to healthy controls, is used for visualization. **c**, Brain and spinal cord regional mapping of differences (T value) between one subtype and all other subtypes using OLS linear models with adjustment for SuStaIn stage. AQP4, aquaporin 4; NMOSD, neuromyelitis optica spectrum disorders; MS, relapsing–remitting multiple sclerosis; NMOSD-C, cortical atrophy leading subtype of AQP4 antibody positive (AQP4 +) NMOSD; NMOSD-SC, spinal cord atrophy leading subtype of AQP4 + NMOSD; NMOSD-CE, cerebellar atrophy leading subtype of AQP4 + NMOSD; MS-C, cortical atrophy leading subtype of MS; MS-SC, spinal cord atrophy subtype of MS; MS-DGM, deep gray matter atrophy subtype of MS

**Fig. 2:**
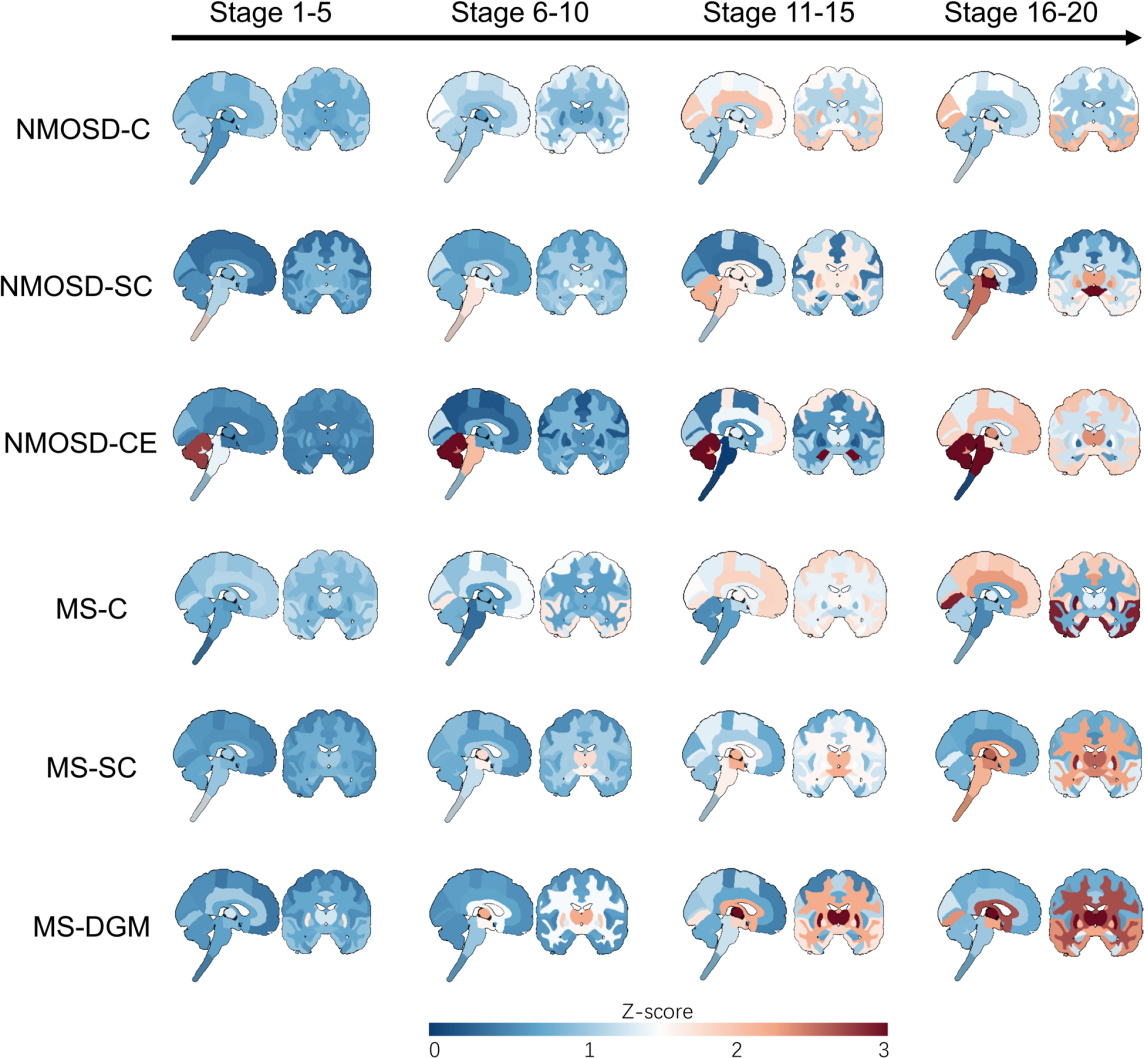
SuStaIn stages of AQP4 + NMOSD and MS subtypes. Progression of each subtype through SuStaIn stages. Each image is a mean of individuals classified for the listed stages. Here stages from 1–20 are displayed, comprising a majority of subtypeable cases (224 out of 268 for MS and 168 out of 171 for AQP4 + NMOSD). AQP4, aquaporin 4; NMOSD, neuromyelitis optica spectrum disorders; MS, relapsing–remitting multiple sclerosis; NMOSD-C, cortical atrophy leading subtype of AQP4 antibody positive (AQP4 +) NMOSD; NMOSD-SC, spinal cord atrophy leading subtype of AQP4 + NMOSD; NMOSD-CE, cerebellar atrophy leading subtype of AQP4 + NMOSD; MS-C, cortical atrophy leading subtype of MS; MS-SC, spinal cord atrophy subtype of MS; MS-DGM, deep gray matter atrophy subtype of MS

Three AQP4 + NMOSD atrophy subtypes were identified (Figs. 1 and 2 and Additional file 1: Fig. S1): (1) cortical atrophy subtype (NMOSD-C, *n* = 87, 31.3%), with gradual atrophy of cortical, subcortical and cerebellar GM, spinal cord and brainstem across all stages; (2) spinal cord atrophy subtype (NMOSD-SC, *n* = 58, 20.9%), with gradual atrophy of the spinal cord, brainstem, subcortical, cerebellar and cortical GM across all stages; and (3) cerebellar atrophy subtype (NMOSD-CE, *n* = 26, 9.4%), with gradual atrophy of cerebellar GM and WM, and brainstem in early stages, and subcortical and cortical GM, cerebral WM and spinal cord in late stages. Additionally, 107 (38.5%) AQP4 + NMOSD were “normal-appearing” (NMOSD-NA).

Three MS atrophy subtypes were also identified (Figs. 1 and 2 and Additional file 1: Fig. S1): (1) MS-C subtype (*n* = 72, 18.4%), with gradual atrophy of cortical and subcortical GM, cerebral WM and brainstem in early stages, and spinal cord, and cerebellum in late stages; (2) MS-SC subtype (*n* = 115, 29.4%), with gradual atrophy of the spinal cord, brainstem, subcortical GM, cerebral and cerebellar WM, cerebral and cerebellar GM across the stages; and (3) Deep gray matter atrophy subtype (MS-DGM, *n* = 81, 20.7%), with gradual atrophy of subcortical GM, cerebral WM and GM, brainstem, cerebellar WM and spinal cord across all stages. Additionally, 123 (31.4%) MS cases were “normal-appearing” (MS-NA).

### Subtype stability and stage progression of AQP4 + NMOSD and MS subtypes

**Fig. 3:**
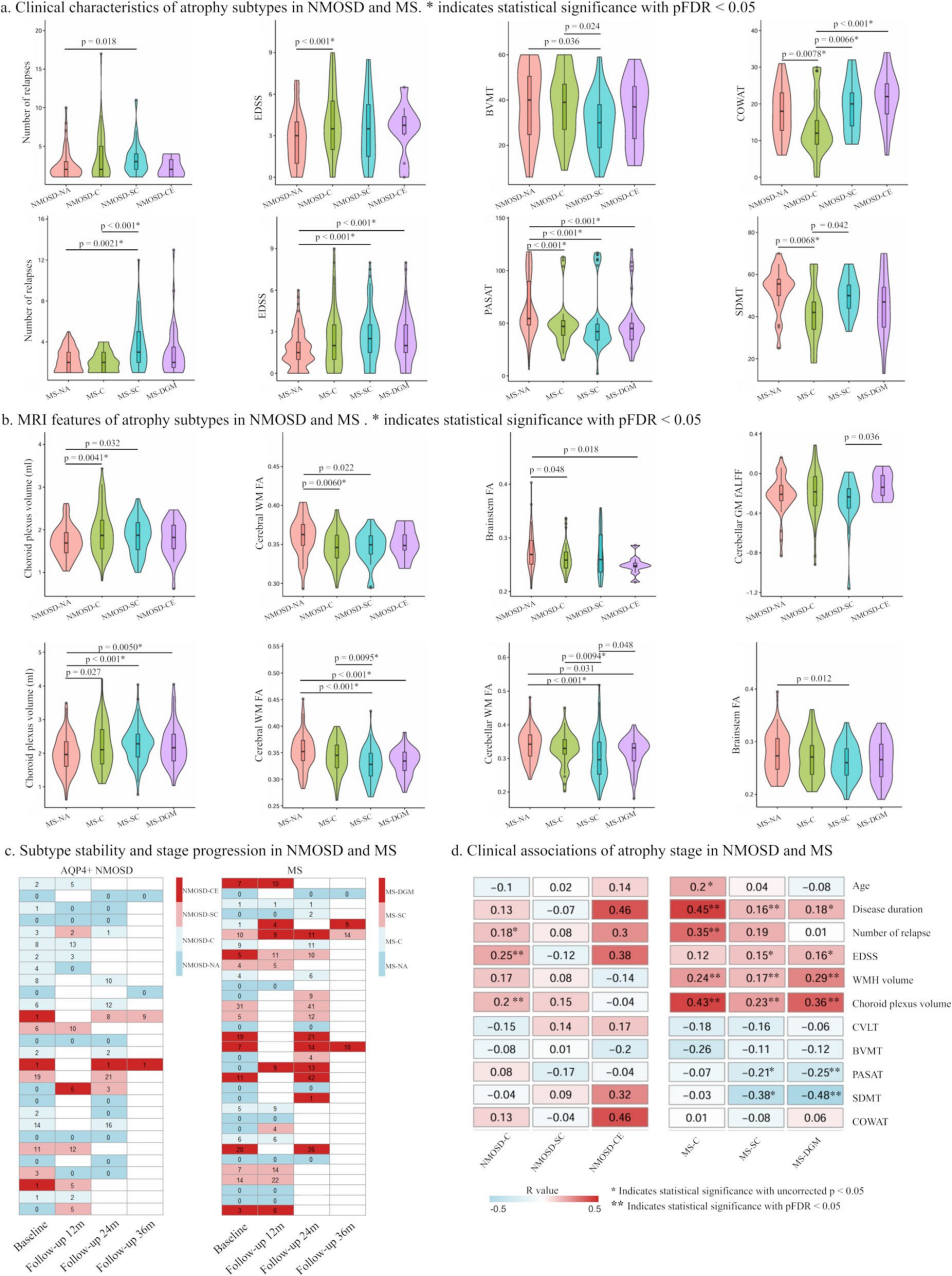
Clinical and MRI characteristics, stability and stage association of AQP4 + NMOSD and MS atrophy subtypes. **a** Clinical characteristics of atrophy subtypes. **b** MRI characteristics of atrophy subtypes. **c** Subtype stability and stage progression of NMOSD and MS subtypes using longitudinal scans. **d** Clinical and MRI associations of atrophy stage. AQP4, aquaporin 4; NMOSD, neuromyelitis optica spectrum disorders; MS, multiple sclerosis; NMOSD-C, cortical atrophy leading subtype of AQP4 antibody positive (AQP4 +) NMOSD; NMOSD-SC, spinal cord atrophy leading subtype of AQP4 + NMOSD; NMOSD-NA, “normal-appearing” AQP4 + NMOSD; NMOSD-CE, cerebellar atrophy leading subtype of AQP4 + NMOSD; MS-NA, “normal-appearing” MS; MS-C, cortical atrophy leading subtype of MS; MS-SC, spinal cord atrophy subtype of MS; MS-DGM, deep gray matter atrophy subtype of MS; WMH, white matter hyperintensity; CVLT, California Verbal Learning Test; Brief BVMT, Visuospatial Memory Test-Revised; PASAT, Paced Auditory Serial Addition Test; SDMT, Symbol Digit Modalities Test; COWAT, Controlled Oral Word Association Test; EDSS, Expanded Disability Status Scale

In AQP4 + NMOSD (Fig. 3c), 20 (20/28, 71.4%) individuals exhibited the same subtype at both baseline and follow-up or progressed from NA to a subtype. Disease stability after excluding individuals classified as NA at baseline and follow-up was found in 80% of cases (12/15).

In MS (Fig. 3c), 30 (30/33, 90.9%) individuals exhibited the same subtype at both baseline and follow-up or progressed from NA to a subtype. Disease stability after excluding individuals classified as NA at baseline and follow-up was found in 84.2% of cases (16/19).

### Clinical and MRI features of AQP4 + NMOSD and MS atrophy subtypes

In AQP4 + NMOSD (Fig. 3a and b), compared with NMOSD-NA, NMOSD-C had higher EDSS score, lower COWAT score, larger choroid plexus volume, and lower FAs of cerebral WM and brainstem. Compared with NMOSD-NA, NMOSD-SC had a higher number of relapses, lower BVMT score, larger choroid plexus volume, and lower cerebral WM-FA. Compared with NMOSD-NA, NMOSD-CE only had lower brainstem FA. In between atrophy subtype comparisons, NMOSD-C showed lower COWAT score than NMOSD-SC and NMOSD-CE. NMOSD-SC showed lower BVMT score than NMOSD-C. NMOSD-CE had higher cerebellar GM-fALFF than NMOSD-SC. Details are found in Additional file 1: Supplementary Results. Additional analyses by stratifying patients according to their primary clinical syndromes were presented in Additional file 1: Fig. S6. Preliminary subgroup analyses showed similar trends with the main findings when the NMOSD patients were stratified by optic neuritis and myelitis, indicating weak associations between these clinical syndromes and specific atrophy subtypes.

In MS (Fig. 3a and b), compared with MS-NA, MS-C had lower PASAT and SDMT scores, and larger choroid plexus volume. Compared with MS-NA, MS-SC had a higher number of relapses, higher EDSS score, lower PASAT score, longer disease duration, larger choroid plexus volume, and lower FAs of cerebral WM, cerebellar WM and brainstem. Compared with MS-NA, MS-DGM had higher EDSS score, lower PASAT score, longer disease duration, larger choroid plexus volume, and lower cerebral and cerebellar WM-FAs. In between atrophy subtype comparisons, MS-SC had a higher number of relapses, and lower cerebral and cerebellar WM-FAs compared with MS-C and MS-DGM, while MS-C had lower SDMT score than MS-SC. Details are found in Additional file 1: Supplementary Results.

### Stage associations in AQP4 + NMOSD and MS atrophy subtypes

In AQP4 + NMOSD, no differences in stages were observed among NMOSD-C, NMOSD-SC and NMOSD-CE. Atrophy stage in NMOSD-C was correlated with EDSS score (R = 0.25, *p* = 0.0031, pFDR = 0.022), number of relapses (R = 0.18, *p* = 0.047, pFDR = 0.21) and choroid plexus volume (R = 0.20, *p* = 0.0072, pFDR = 0.041) (Fig. 3d).

In MS, no differences in stages were observed among MS-C, MS-SC and MS-DGM. Atrophy stage in MS-C was correlated with age (R = 0.20, *p* = 0.014, pFDR = 0.39), disease duration (R = 0.45, *p* < 0.0001, pFDR < 0.0001), relapse (R = 0.35, *p* = 0.0026, pFDR = 0.0095), WMH volume (R = 0.24, *p* = 0.0027, pFDR =  = 0.0095) and choroid plexus volume (R = 0.43, *p* < 0.0001, pFDR < 0.0001). Atrophy stage in MS-SC was correlated with disease duration (R = 0.16, *p* = 0.014, pFDR = 0.039), EDSS score (R = 0.15, *p* = 0.022, pFDR = 0.057), PASAT score (R = −0.21, *p* = 0.032, pFDR = 0.074), SDMT score (R = −0.38, *p* = 0.035, pFDR = 0.079), WMH volume (R = 0.17, *p* = 0.0063, pFDR = 0.020) and choroid plexus volume (R = 0.23, *p* = 0.00036, pFDR = 0.0014). Atrophy stage in MS-DGM was correlated with disease duration (R = 0.18, *p* = 0.026, pFDR = 0.065), EDSS score (R = 0.16, *p* = 0.041, pFDR = 0.088), PASAT score (R = −0.25, *p* = 0.014, pFDR = 0.039), SDMT score (R = −0.48, *p* = 0.0044, pFDR = 0.015), WMH volume (R = 0.29, *p* = 0.00020, pFDR = 0.00085) and choroid plexus volume (R = 0.36, *p* < 0.0001, pFDR < 0.0001) (Fig. 3d).

### Disability worsening and relapse of AQP4 + NMOSD and MS atrophy subtypes

**Fig. 4:**
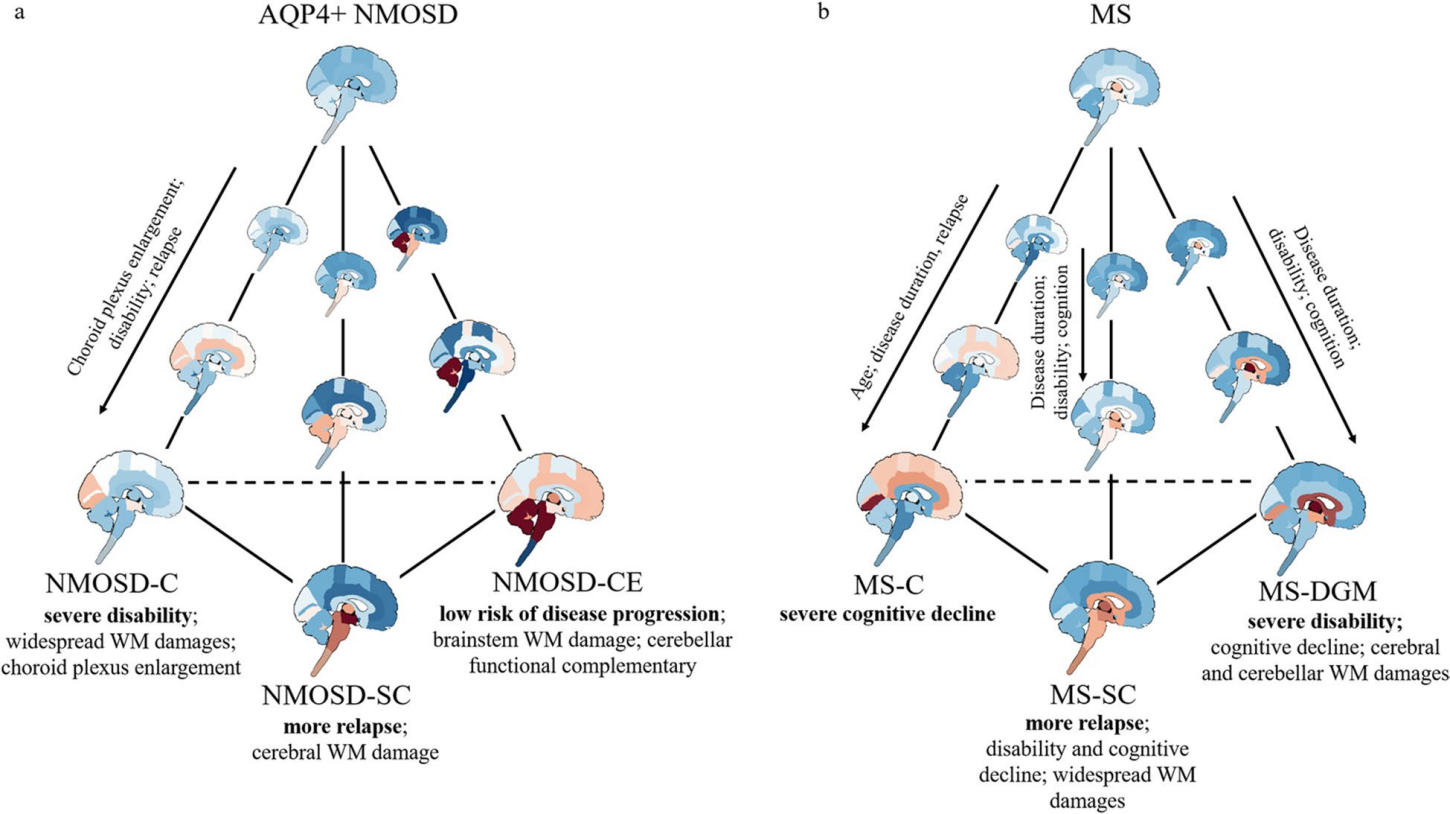
A theoretical model summarizing brain and spinal cord atrophy subtypes in AQP4 + NMOSD (**a**) and MS (**b**). Atrophy varies along the axis of disability, relapse, cognition decline, age and disease duration (vertical axis in the diagram) in different AQP4 + NMOSD and MS subtypes. Atrophy varies along a spatiotemporal dimension (horizontal axis in the diagram), such that an individual can be described by their fit along one of at least three trajectories. The text indicates the clinical characteristics of each subtype. The text in bold reflects major clinical differences between subtypes, while normal text reflects MR-related characteristics that differentiate subtypes from normal-appearing individuals. AQP4, aquaporin 4; NMOSD, neuromyelitis optica spectrum disorders; MS, multiple sclerosis; NMOSD-C, cortical atrophy leading subtype of AQP4 antibody positive (AQP4 +) NMOSD; NMOSD-SC, spinal cord atrophy leading subtype of AQP4 + NMOSD; NMOSD-CE, cerebellar atrophy leading subtype of AQP4 + NMOSD; MS-C, cortical atrophy leading subtype of MS; MS-SC, spinal cord atrophy subtype of MS; MS-DGM, deep gray matter atrophy subtype of MS; WM, white matter

Here, we reported the step-wise backward Cox regression findings (final model by “autoReg” package in R, see Table 2 for details). In AQP4 + NMOSD, NMOSD-CE showed relatively reduced EDSS progression (Hazard Ratio [HR] = 0.11, 95%CI [0.01, 0.99], *p* = 0.049) and relapse (HR = 0.13 [0.03, 0.69], *p* = 0.017). In MS, a late stage had a slightly increased risk of disease phenotype conversion from relapsing–remitting MS to SPMS (HR = 1.03, [1.00, 1.06], *p* = 0.045). No association of disease subtypes, stages, age or sex was observed for the follow-up EDSS worsening or relapse of MS. A summary of AQP4 + NMOSD and MS atrophy subtypes were provided in Fig. 4.

**Table 2:** Univariate and multivariate cox proportional-hazards regression for follow-up disability worsening and relapse in NMOSD and MS

	Variable	HR (univariable, CI, p)	HR (multivariable, CI, p)	HR (final, CI, p)
AQP4 + NMOSD				
EDSS progression	NMOSD-C	0.71 (0.31–1.64, *p* = 0.43)	0.57 (0.19–1.69, *p* = 0.31)	
	NMOSD-SC	1.29 (0.54–3.10, *p* = 0.57)	0.43 (0.12–1.51, *p* = 0.19)	
	NMOSD-CE	0.23 (0.03–1.77, *p* = 0.16)	**0.07 (0.01–0.85, *p* = 0.037)**	**0.11 (0.01–0.99, *p* = 0.049)**
	Atrophy stage	1.01 (0.92–1.12, *p* = 0.79)	1.08 (0.96–1.22, *p* = 0.22)	
	Age (years)	1.02 (0.99–1.06, *p* = 0.12)	1.01 (0.98–1.05, *p* = 0.50)	
	Sex (Female)	0.60 (0.18–2.05, *p* = 0.42)	0.34 (0.07–1.53, *p* = 0.16)	0.30 (0.08–1.09, *p* = 0.068)
	Disease duration (months)	0.99 (0.99–1.00, *p* = 0.12)	0.99 (0.99–1.00, *p* = 0.097)	0.99 (0.99–1.00, *p* = 0.077)
Follow-up relapse	NMOSD-C	1.23 (0.66–2.29, *p* = 0.52)	1.30 (0.53–3.16, *p* = 0.57)	
	NMOSD-SC	0.72 (0.31–1.63, *p* = 0.43)	0.45 (0.16–1.26, *p* = 0.13)	0.44 (0.18–1.07, *p* = 0.070)
	NMOSD-CE	0.39 (0.09–1.68, *p* = 0.21)	**0.14 (0.03–0.78, *p* = 0.024)**	**0.13 (0.03–0.69, *p* = 0.017)**
	Atrophy stage	1.01 (0.93–1.10, *p* = 0.78)	1.03 (0.93–1.14, *p* = 0.58)	
	Age (years)	0.99 (0.97–1.02, *p* = 0.54)	**0.97 (0.94–1.00, *p* = 0.030)**	**0.97 (0.94–1.00, *p* = 0.032)**
	Sex (Female)	0.31 (0.09–1.05, *p* = 0.059)	**0.12 (0.03–0.49, *p* = 0.0030)**	**0.14 (0.04–0.53, *p* = 0.004)**
	Disease duration (months)	1.00 (0.99–1.00, *p* = 0.37)	**0.99 (0.99–1.00, *p* = 0.048)**	1.00 (0.99–1.00, *p* = 0.080)
MS				
EDSS progression	MS-C	0.82 (0.42–1.61, *p* = 0.57)	0.72 (0.27–1.92, *p* = 0.51)	
	MS-SC	1.23 (0.74–2.03, *p* = 0.43)	0.87 (0.38–1.96, *p* = 0.73)	
	MS-DGM	0.91 (0.47–1.75, *p* = 0.77)	0.78 (0.31–1.98, *p* = 0.60)	
	Atrophy stage	1.01 (0.99–1.04, *p* = 0.29)	1.01 (0.98–1.04, *p* = 0.46)	
	Age (years)	1.00 (0.97–1.02, *p* = 0.69)	0.99 (0.97–1.02, *p* = 0.65)	
	Sex (Female)	1.17 (0.69–1.96, *p* = 0.56)	1.17 (0.68–2.00, *p* = 0.58)	
	Disease duration (months)	1.00 (1.00–1.01, *p* = 0.14)	1.00 (1.00–1.01, *p* = 0.25)	1.00 (1.00–1.01, *p* = 0.14)
SPMS conversion	MS-C	1.14 (0.49–2.63, *p* = 0.77)	0.99 (0.22–4.53, *p* = 0.99)	
	MS-SC	1.30 (0.65–2.64, *p* = 0.46)	0.91 (0.23–3.62, *p* = 0.90)	
	MS-DGM	0.84 (0.32–2.20, *p* = 0.72)	0.78 (0.17–3.61, *p* = 0.75)	
	Atrophy stage	**1.04 (1.01–1.07, *p* = 0.0070)**	**1.03 (1.00–1.07, *p* = 0.047)**	**1.03 (1.00–1.06, *p* = 0.045)**
	Age (years)	1.01 (0.97–1.04, *p* = 0.74)	1.00 (0.96–1.04, *p* = 0.99)	
	Sex (Female)	1.11 (0.53–2.30, *p* = 0.79)	0.92 (0.43–1.97, *p* = 0.83)	
	Disease duration (months)	**1.01 (1.00–1.01, *p* = 0.0090)**	1.01 (1.00–1.01, *p* = 0.067)	1.01 (1.00–1.01, *p* = 0.065)
Follow-up relapse	MS-C	0.80 (0.37–1.72, *p* = 0.56)	0.95 (0.33–2.78, *p* = 0.93)	
	MS-SC	1.06 (0.58–1.93, *p* = 0.85)	1.12 (0.40–3.07, *p* = 0.83)	
	MS-DGM	1.31 (0.67–2.56, *p* = 0.43)	1.12 (0.41–3.09, *p* = 0.83)	
	Atrophy stage	1.02 (0.99–1.05, *p* = 0.31)	1.00 (0.97–1.04, *p* = 0.83)	
	Age (years)	1.00 (0.97–1.02, *p* = 0.82)	0.99 (0.96–1.02, *p* = 0.61)	
	Sex (Female)	0.88 (0.44–1.74, *p* = 0.71)	0.72 (0.33–1.59, *p* = 0.42)	
	Disease duration (months)	1.00 (1.00–1.01, *p* = 0.064)	1.00 (1.00–1.01, *p* = 0.13)	1.00 (1.00–1.01, *p* = 0.064)

Univariate, multivariate and final cox proportional-hazards regression models were fitted using “autoReg” in R (the final model indicates the step-wise backward regression). HRs were presented with 95% confidence intervals. Bold text indicated statistical significance in univariate, multivariate or final regression models

*HR* Hazard ratio, *AQP4* + *NMOSD* AQP4 antibody positive neuromyelitis optica spectrum disorders, *MS* Multiple sclerosis, *EDSS* Expanded Disability Status Scale, *SPMS* Secondary progressive MS, *NMOSD-C* Cortical atrophy leading subtype of AQP4 + NMOSD, *NMOSD-SC* Spinal cord atrophy leading subtype of AQP4 + NMOSD, *NMOSD-CE* Cerebellar atrophy leading subtype of AQP4 + NMOSD, *MS-C* Cortical atrophy leading subtype of MS, *MS-SC* Spinal cord atrophy subtype of MS, *MS-DGM* Deep gray matter atrophy subtype of MS

### Treatment response to DMT among AQP4 + NMOSD and MS atrophy subtypes

**Fig. 5:**
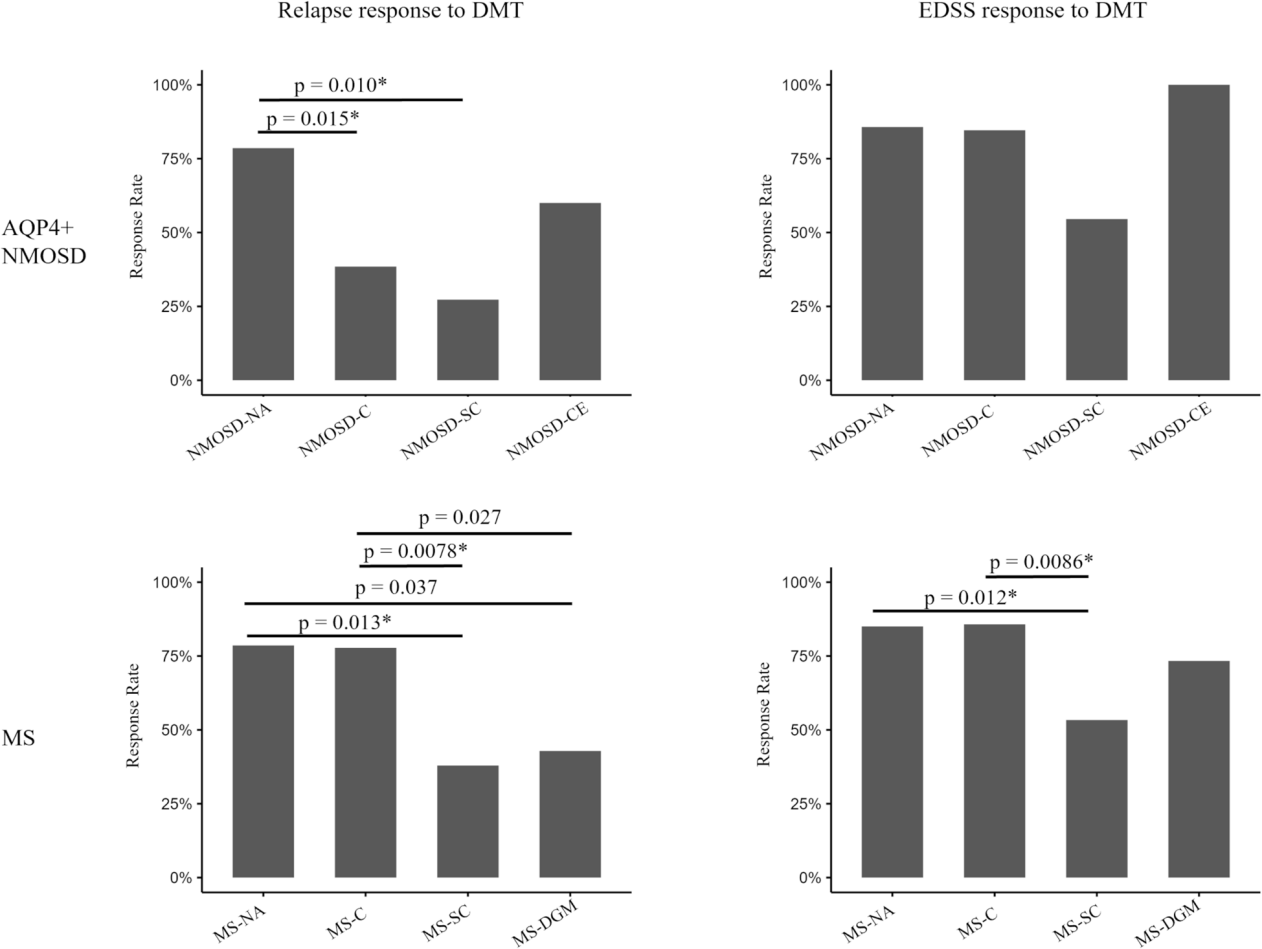
Treatment response to DMT regarding the disease relapse and physical disability worsening (EDSS worsening) among AQP4 + NMOSD and MS atrophy subtypes. AQP4, aquaporin 4; NMOSD, neuromyelitis optica spectrum disorders; MS, multiple sclerosis; NMOSD-NA, “normal-appearing” AQP4 antibody positive (AQP4 +) NMOSD; NMOSD-C, cortical atrophy leading subtype of AQP4 + NMOSD; NMOSD-SC, spinal cord atrophy leading subtype of AQP4 + NMOSD; NMOSD-CE, cerebellar atrophy leading subtype of AQP4 + NMOSD; MS-NA, “normal-appearing” MS; MS-C, cortical atrophy leading subtype of MS; MS-SC, spinal cord atrophy subtype of MS; MS-DGM, deep gray matter atrophy subtype of MS; DMT, disease-modifying therapy; EDSS, Expanded Disability Status Scale

For response to DMT regarding relapse, in AQP4 + NMOSD (Fig. 5), response rates were 78.6% (11/14) for NMOSD-NA, 38.5% (10/26) for NMOSD-C, 27.3% (3/11) for NMOSD-SC and 60.0% (3/5) for NMOSD-CE. NMOSD-C (*p* = 0.015, pFDR = 0.046) and NMOSD-SC (*p* = 0.010, pFDR = 0.046) had lower response rates compared with NMOSD-NA. In MS, response rates were 78.6% (11/14) for MS-NA, 77.8% (14/18) for MS-C, 37.9% (11/29) for MS-SC and 42.9% (9/21) for MS-DGM. MS-SC had a lower response rate than MS-NA (*p* = 0.013, pFDR = 0.038) and MS-C (*p* = 0.0078, pFDR = 0.038). MS-DGM had a lower response rate compared with MS-NA (*p* = 0.037, pFDR = 0.055) and MS-C (*p* = 0.027, pFDR = 0.054).

For response to DMT regarding EDSS worsening, in AQP4 + NMOSD (Fig. 5), response rates were 85.7% (12/14) for NMOSD-NA, 84.6% (22/26) for NMOSD-C, 54.5% (6/11) for NMOSD-SC and 100% (5/5) for NMOSD-CE. No statistical difference among AQP4 + NMOSD subtypes was observed. In MS, response rates were 85% (17/20) for MS-NA, 85.7% (18/21) for MS-C, 53.3% (32/60) for MS-SC and 73.3% (22/30) for MS-DGM. MS-SC had a lower response rate than MS-NA (*p* = 0.012, pFDR = 0.035) and MS-C (*p* = 0.0086, pFDR = 0.035).

## Discussion

In this study, we analyzed the spatiotemporal pattern of CNS atrophy in patients with AQP4 + NMOSD and MS using advanced machine learning techniques. We found that around 60% of AQP4 + NMOSD patients and 70% of MS patients exhibited CNS atrophy. Among NMOSD patients with CNS atrophy, subtypes included NMOSD-C with severe disability and cognitive decline, NMOSD-SC with a high number of relapses, and NMOSD-CE with low disease progression risk. In MS, MS-C showed severe cognitive decline, MS-SC had enhanced relapses and disability, and MS-DGM exhibited severe physical disability and cognitive decline. Cortical and spinal cord atrophy subtypes were shared between AQP4 + NMOSD and MS, but they were characterized by distinct clinical and MRI features. A cerebellar atrophy subtype was specific to AQP4 + NMOSD, whereas a subcortical atrophy subtype was specific to MS.

CNS atrophy is a known feature of MS, and recent studies have also identified it in NMOSD [7, 10, 19]. Our research focused on a large cohort of AQP4 + NMOSD patients, revealing variability in CNS changes. Notably, some AQP4 + NMOSD and MS patients without apparent CNS atrophy had disease relapses and disabilities similar to those with atrophy. Furthermore, patients with"normal-appearing"brain scans, despite lacking significant atrophy, had lower cognitive function scores than HCs, though their scores were better than those with CNS atrophy. These findings indicate that the absence of visible CNS atrophy does not guarantee preserved cognitive function, aligning with the well-known clinico-radiological paradox in NMOSD and MS [30]. This paradox reflects the discrepancy between radiological abnormalities and clinical symptoms, where significant structural or functional damage may not always manifest as observable clinical deficits, and conversely, severe symptoms may occur in the absence of detectable MRI abnormalities. Such discrepancies underscore the need for multimodal approaches, combining structural, diffusion, and functional MRI data, to fully elucidate the neurobiological underpinnings of cognitive and physical disability in these diseases. Previous studies have shown that reduced altered structural and functional connectivity, can correlate with cognitive dysfunction [31–34]. Future studies integrating diffusion and functional MRI data with SuStaIn modeling could provide additional insights into the microstructural and functional network changes that contribute to cognitive decline, particularly in patients with ‘normal-appearing’ structural scans.

The atrophy subtypes of AQP4 + NMOSD have multiple implications for interpreting NMOSD heterogeneity. This study revealed marked imaging, clinical and prognostic heterogeneity in AQP4 + NMOSD, consistent with previous studies [1, 35]. As a novel feature, a cortical atrophy subtype of AQP4 + NMOSD was identified. It seems that one of the underlying contributors to cortical subtype is WM microstructural damage [36–38]. Enlarged choroid plexus volume, a hallmark of neuroinflammation [39–41], suggests diffuse brain neuroinflammation underlying cortical atrophy in this subtype [42–44]. Larger choroid plexus volume, enhanced relapse and more severe disability at the late stage of NMOSD-C may further support the underlying neuroinflammation and damage. Spinal cord damage has been commonly highlighted in studies on NMOSD [14, 22]. In this atrophy subtype, we also observed enlarged choroid plexus, indicating neuroinflammation as a shared underlying pathology in NMOSD-SC and NMOSD-C. Consistent with our previous findings, the spinal cord showed enhanced disease relapse in NMOSD-SC, which was different from NMOSD-C that seemed to be more associated with the disease course (disease stage) [14]. Cerebellar alteration has been considered a characterizing feature in AQP4 + NMOSD in our previous studies, which could be used for differential diagnosis [7, 8]. The NMOSD-CE subtype presented with only brainstem WM damage and local functional complementary, which implies the mildest CNS damage in this subtype [8]. As expected, a low risk of follow-up disability and relapse was observed in NMOSD-CE. These findings may provide a novel perspective to reveal the heterogeneity of AQP4 + NMOSD in addition to the clinical subtypes (e.g., myelitis and optic neuritis).

Previous reports consistently considered severe cortical atrophy as a hallmark of MS [15, 17, 18]. The MS-C subtype was characterized by severe cognitive decline, which may be mainly attributed to diffuse chronic neuroinflammation during the disease course as reflected by progressive enlargement of choroid plexus [45–47]. Spinal cord atrophy has also been widely reported in MS [13, 14, 48]. This subtype showed diffuse brain white matter damage, indicating an interaction between spinal cord and brain axonal damages [49]. Similar to AQP4 + NMOSD, the MS-SC subtype was highly associated with relapse, corroborating a previous study [50]. Spinal cord atrophy also accounted for physical disability in MS during the disease course, consistent with previous findings [13, 14, 48]. DGM atrophy, a highlighted marker of MS, is associated with both cognitive decline and physical disability [17, 18]. MRI and clinical features were similar to those of the MS-SC subtype, while MS-SC involves relapse, which was absent in this subtype [51]. None of MS subtypes plays a role in disease progression, while later stages had a high risk of SPMS conversion, together with the association of stage and disease duration, indicating a natural gradual progression during the disease course [52].

The findings suggest that cortical atrophy is a common subtype in AQP4 + NMOSD and MS, potentially driven by neuroinflammation, especially in later disease stages. In AQP4 + NMOSD, this subtype is linked to physical disability, while in MS, it correlates with cognitive impairment. Spinal cord atrophy is prevalent in both conditions and is associated with relapse, though it presents different white matter microstructural damage patterns and has a lesser impact on disability in AQP4 + NMOSD compared to MS. Cerebellar atrophy is unique to AQP4 + NMOSD, exhibiting mild brain damage and favorable prognosis. Conversely, subcortical atrophy is specific to MS and is associated with both physical disability and cognitive decline, highlighting its significance in MS pathology. Specifically, the atrophy trajectory among AQP4 + NMOSD patients across various atrophy subtypes sheds light on potential patterns of disease progression, offering a novel perspective for understanding the subtle brain tissue damage in AQP4 + NMOSD patients [10, 53]. These findings provide insights into atrophy patterns in both conditions, which can inform clinical decision-making and aid in stratified medicine approaches. Longitudinal analysis indicates that these subtypes remain stable over time and are intrinsic to cognitive decline, physical disability, and disease progression. This research also identifies potential therapeutic targets for different subtypes to mitigate atrophy and improve patient outcomes.

There were some limitations in this study. First, this study utilized retrospective and prospective multicenter data to boost the sample size to develop a statistically robust subtyping model for NMOSD and MS. The sex ratio is different between HC and disease groups, which may bias the neuroimaging findings, even we used “MatchIt” to match the sex. More strictly age- and sex-matched samples and further external validations are needed to confirm the atrophy subtypes identified in this study. Secondly, this study focused on both brain and spinal cord atrophy subtypes with robust structural metrics derived from high-resolution 3D T1-weighted images. Adding other potential pathologies, including demyelinating lesions and diffuse WM changes into SuStaIn models might provide added value in determining disease heterogeneity. Furthermore, the heterogeneity of mixed clinical presentations may limit the statistical power of atrophy subtype analyses, particularly in AQP4 + NMOSD subgroup analysis. Future studies with larger cohorts are needed to validate these findings. Lastly, whether the different treatments contribute to the atrophy subtypes and whether disease-subtype-tailored treatments are efficient to delay brain and spinal cord atrophy and disease progression in similar atrophy subtypes of AQP4 + NMOSD and MS need further investigation in future clinical trials with standardized treatments.

## Conclusions

Using a machine-learning approach, both shared and distinct spatiotemporal brain and spinal cord atrophy subtypes were explored in AQP4 + NMOSD and MS, with distinct clinical and MRI features. The preliminarily identified AQP4 + NMOSD and MS subtypes may provide a better interpretation of disease heterogeneity and help develop targeted management strategies.

## Supplementary Information

Additional file 1: Supplementary Method; Supplementary Results; Figures S1-S6. FigS1 - [A flowchart of the subjects included in this study]. FigS2 – [The case distribution of the multicenter dataset]. FigS3 – [Selection of the optimal number of subtypes by SuStaIn, and subtypes and stages of AQP4+ NMOSD and MS]. FigS4– [The clinical and cognitive characteristics of the “normal appearing” AQP4+ NMOSD and MS patients compared to age- and sex- matched HCs and those with CNS atrophy]. FigS5 – [The MR characteristics of the “normal appearing” AQP4+ NMOSD and MS patients compared to age- and sex- matched HCs and those with CNS atrophy]. FigS6 – [The potential association analysis of atrophy subtypes and clinical syndrome in AQP4+ NMOSD]; Table S1 – [Details of the MR protocols].

## Data Availability

The code and source data are available upon the requirement received by the corresponding author.

## Abbreviations

AQP4 + NMOSD: Aquaporin-4 antibody positive neuromyelitis optica spectrum disorders
BVMT: Brief Visuospatial Memory Test
COWAT: Controlled Oral Word Association Test
CVLT: California Verbal Learning Test
EDSS: Expanded Disability Status Scale
FLAIR: Fluid-attenuated inversion recovery
GMV: Gray matter volume
HC: Healthy control
MS: Multiple sclerosis
MUCCA: Mean upper cervical cord area
PASAT: Paced Auditory Serial Addition Test
SuStaIn: Subtype and Stage Inference
SDMT: Symbol Digit Modalities Test
SPMS: Secondary progressive MS
T1W: T1-weighted
TIV: Total intracranial volume
WMH: White matter hyperintensity
