# Supplementary material for: Spatiotemporal subtypes of brain and spinal cord atrophy in neuromyelitis optica spectrum disorders and multiple sclerosis
Source: BMC Med. 2025 Sep 2;23:514. doi: 10.1186/s12916-025-04366-7 (PMC12403954; doi:10.1186/s12916-025-04366-7)
Supplement: Supplementary file 1 — Additional file 1: Supplementary Method; Supplementary Results; Figures S1-S6. FigS1 - [A flowchart of the subjects included in this study]. FigS2 – [The case distribution of the multicenter dataset]. FigS3 – [Selection of the optimal number of subtypes by SuStaIn, and subtypes and stages of AQP4+ NMOSD and MS]. FigS4– [The clinical and cognitive characteristics of the “normal appearing” AQP4+ NMOSD and MS patients compared to age- and sex- matched HCs and those with CNS atrophy]. FigS5 – [The MR characteristics of the “normal appearing” AQP4+ NMOSD and MS patients compared to age- and sex- matched HCs and those with CNS atrophy]. FigS6 – [The potential association analysis of atrophy subtypes and clinical syndrome in AQP4+ NMOSD]; Table S1 – [Details of the MR protocols]. [file 12916_2025_4366_MOESM1_ESM.docx]

Supplementary Materials

Supplementary Methods

Cognitive tests

Cognitive tests included California Verbal Learning Test, Third Edition (CVLT-III) for the evaluation of verbal learning and memory, Brief Visuospatial Memory Test-Revised (BVMT-R) for the assessment of optical recognition deficits and their progression, Paced Auditory Serial Addition Test (PASAT, 3 seconds) for the evaluation of auditory information processing speed and flexibility, as well as calculation ability, Symbol Digit Modalities Test (SDMT) for the assessment of neurological dysfunction underpinned by attention, perceptual speed, motor speed, and visual scanning, and Controlled Oral Word Association Test (COWAT) for the evaluation of verbal fluency.

MR image processing

Prior to MR image processing, all MR images were reviewed by an experienced radiologist (X.X., eight years in radiology) for quality control regarding artifacts, contrast, and signal-to-noise ratio. In clinical settings, white matter hyperintensities (which are white matter lesions) in multiple sclerosis and its mimics, such as NMOSD, are particularly visible on MR images, especially on T2-FLAIR images. While FLAIR images are typically acquired via 2D image acquisition in clinical settings for rapid acquisition, in this study, we selected FLAIR images with a slice thickness of less than 4mm and an in-plane resolution of less than 1mm to minimize the partial volume effect on white matter lesions. Additionally, to ensure the quality of lesion filling, we excluded images with obvious artifacts (e.g., movement or aliasing artifacts), low signal-to-noise ratio, or low tissue contrast for both 3D T1W and FLAIR images to achieve high-quality lesion and brain tissue segmentations.

White matter hyperintensity (WMH) was segmented, and WMH volumes were determined using a home-developed WMH segmentation model for fluid attenuated inversion recovery (FLAIR) images. Details could be found in our published work and the model could be obtained via https://github.com/lchdl/DeepWMH. The segmentations were checked and modified if necessary by Y.D. (a radiologist with 14 years of experience in neuroradiology) using ITK-SNAP (version 3.8.0, http://www.itksnap.org).

To address the issue of lesion filling in 3D T1-weighted (T1W) images, we initially realigned and resampled the FLAIR image and its associated lesion mask to the space of the corresponding 3D T1W image. Subsequently, we utilized the "lesion_filling" command within the FSL (https://fsl.fmrib.ox.ac.uk/fsl/fslwiki/lesion_filling) to execute the lesion filling process. Lesion-filled 3D T1-weighted (T1W) images were then preprocessed using FreeSurfer (version 7.3.2, <http://surfer.nmr.mgh.harvard.edu/>). First, the “recon-all” pipeline with default parameters was used to extract the cortical surface and subcortical regions. Then, the segmented volumes and created surfaces were visually inspected for misclassification during the reconstruction process by X.X.

Additionally, mean upper cervical cord areas (MUCCAs) were calculated at C1 to C3 levels on 3D T1W images to assess potential spinal cord atrophy using the Spinal Cord Toolbox (version 6.3, https://spinalcordtoolbox.com/). The 3D T1W sagittal images were acquired for comprehensive coverage of the whole brain and extended to include the cervical spinal cord, at least from the C1 to C3 vertebrae. First, the “sct_deepseg_sc”, which is a fully automated spinal cord segmentation method based on a deep learning convolutional neural network module that is incorporated in the Spinal Cord Toolbox, was used to segment spinal cord images. Then “sct_label_vertebrae” was used with default settings to label vertebras. Last, “sct_process_segmentation” was used to segment vertebras and calculate their morphological parameters. During image processing, spinal cord segmentation and labels were visually checked by X.X.

The choroid plexus was segmented using a home-developed deep learning algorithm with 3D nnU-Net based on 3D T1W images. The detailed nnU-Net architecture could be found in previous study. For our network, an input patch size of 16×320×320 was selected. Downsampling is done with strided convolutions, upsampling is implemented as convolution transposed. Feature map sizes are displayed in the encoder part of the architecture. The feature maps in the decoder mirror the encoder. The feature map sizes are: 16×320×320, 16×160×160, 16×80×80, 16×40×40, 8×20×20, 4×10×10, and 4×5×5. We used the maximum Dice score in the validation set as the criteria to select the optimal model. First, the choroid plexuses on 3D T1W images of 300 randomly selected healthy controls (HCs, aged from 6 to 90 years) and 300 patients with multiple neurological diseases by various MR scanners (GE Premier and Discovery750, Philips Ingenia DNA and CX, and Siemens Prisma and Verio) in Beijing Tiantan Hospital were manually labeled by two neuroradiologists (L.Q. with 7 years of experience in neuroradiology and J.L. with 6 years of experience in neuroradiology). The labeled choroid plexus was further reviewed and modified (if necessary) by another neuroradiologist (Y.D.). Subsequently, 420 randomly selected cases were employed for 3D nnU-Net development (training [n = 360] and validation [n = 60]), and the remaining 180 cases were utilized for model testing (Dice score = 0.84). Finally, the developed deep learning model was applied to segment all the T1W images used in this study.

Diffusion image processing was carried out with FMRIB’s Diffusion Toolbox (FDT, FSL 6.0.1, www.fmrib.ox.ac.uk/fsl). First, all diffusion images were visually assessed, and images with poor quality (e.g., significant artifacts or incomplete FOV) were excluded. Diffusion images were preprocessed, including image distortion correction in case a B0 image with reverse phase-encoding (if the B0 image with reverse phase-encoding was not acquired, a Synthesized B0 image for distortion correction using the Synb0-DisCo approach was obtained), eddy-current and motion artifact correction, and skull removal. Finally, fractional anisotropy (FA) was calculated based on the diffusion tensor model for each voxel. For each subject, the B0 image was first co-registered to 3D T1W image by affine transformation. Then all FA maps were wrapped into the Montreal Neurological Institute (MNI) space and resampled into isotropic 3 mm using the transformation matrices of B0 to 3D T1W and 3D T1W to MNI space.

Resting state functional MRI images (rs-fMRIs) were processed using DPABI (Data Processing & Analysis for (Resting-State) Brain Imaging, Version 7.0, http://www.rfmri.org/dpabi). First, all rs-fMRI images were visually checked, and images with poor quality (e.g., significant artifacts or incomplete FOV) were excluded. Discarding the first 10 volumes, realigning rs-fMRI volumes, regressing covariates (including the linear trend, 24 motion-related parameters, and mean signals within CSF and white matter), registering T1W and rs-fMRI images into the MNI space were performed. rs-fMRI volumes were resampled into isotropic 3 mm. Participants with more than 3 mm maximum displacement in the x y or z axis, or 3° of angular motion during the rs-fMRI realigning were excluded in the following parameter calculation. Fractional amplitude of low frequency fluctuation (fALFF with 0.01-0.1Hz) was firstly calculated with normalized rs-fMRI volume. All the above calculated rs-fMRI parameter maps were transformed into z-scores to create standardized subject-level maps and smoothed by a Gaussian kernel with full width at half maximum of 4 mm.

Supplementary Results

Details of Clinical and MRI features of AQP4+ NMOSD and MS subtypes

For AQP4+ NMOSD, no difference in age, education or disease duration was observed between subtypes. NMOSD-CE (81%, 21/26) showed lower female ratio than other subtypes (NMOSD-C [94%, 82/87] and NMOSD-SC [90%, 52/58]). Compared with NMOSD-NA, NMOSD-C showed higher EDSS score (p < 0.001 [false discovery rate, FDR-adjusted p = 0.0040]) and lower COWAT score (p = 0.0078 [0.016]). NMOSD-SC showed elevated relapse (p = 0.018 [0.11]) and lower BVMT (p = 0.036 [0.11]). In between subtype comparison, NMOSD-C had lower COWAT compared to NMOSD-SC (p = 0.0066 [0.016]) and NMOSD-CE (p < 0.001 [0.0023]). NMOSD-SC showed lower BVMT than NMOSD-C (p = 0.024 [0.11]). No difference in WMH volume was observed between AQP4+ NMOSD subtypes. Compared with NMOSD-NA, NMOSD-C had larger choroid plexus volume (p = 0.0041 [p = 0.025]), and lower cerebral and brainstem WM-FA (p = 0.0060 [0.036] and 0.048 [0.14], respectively). NMOSD-SC had larger choroid plexus volume (p = 0.032 [0.096]) and lower cerebral WM-FA (p = 0.022 [0.065]). NMOSD-CE had lower brainstem FA (p = 0.018 [0.11]). NMOSD-CE had higher cerebellar GM-fALFF than NMOSD-SC (p = 0.036 [0.21]).

In one-versus-all comparisons in AQP4+ NMOSD, NMOSD-C had lower COWAT (p = 0.045 [1]) and higher choroid plexus volume (p = 0.034 [1]). NMOSD-SC had lower choroid plexus volume (p = 0.045 [1]) and higher SDMT (p = 0.039 [1]).

In MS, no differences in age and sex were observed between MS subtypes. Compared with MS-NA, MS-C had lower PASAT (p < 0.001 [0.0017]) and SDMT (p = 0.0068 [0.041]). MS-SC had longer disease duration (p = 0.0077 [0.023]), elevated baseline relapse (p = 0.0021 [0.0062]), higher EDSS (p < 0.001 [< 0.001]), and lower PASAT (p < 0.001 [< 0.001]). MS-DGM had longer disease duration (p = 0.00094 [0.0056]), higher EDSS (p < 0.001 [0.0021]) and lower PASAT (p < 0.001 [< 0.001]). In between subtype comparisons, MS-SC had elevated baseline relapse than MS-C (p < 0.001 [0.0058]), while MS-C had lower SDMT than MS-SC (p = 0.042 [0.13]). Compared with MS-NA, MS-C had larger choroid plexus volume (p = 0.027 [0.055]). MS-SC had larger choroid plexus volume (p < 0.001 [< 0.001]), and lower cerebral, cerebellar and brainstem WM-FA (p < 0.0001 [< 0.001], < 0.001 [< 0.001] and 0.012 [0.072]). MS-DGM had larger choroid plexus volume (p = 0.0050 [0.015]), lower cerebral and cerebellar WM-FA (p < 0.001 [0.0026] and 0.031 [0.061], respectively). MS-SC showed lower cerebral WM-FA than MS-C (p = 0.0095 [0.019]) and cerebellar WM-FA than MS-DGM (p = 0.048 [0.072]) and MS-C (p = 0.0094 [0.028]).

In one-versus-all comparisons in MS, MC-C had higher FA values for cerebral WM (p = 0.014 [0.51]) and brainstem (p < 0.001 [0.027]). MS-SC had lower brainstem FA (p < 0.001 [< 0.001]). MS-DGM had higher WMH volume (p < 0.001 [0.030]).

Additional findings on the associations of disease stage in AQP4+ NMOSD with MS subtypes

After adjustment for education level (cognitive score), the following findings were obtained. No correlation was observed in NMOSD-C, NMOSD-SC and NMOSD-CE. In MS-SC, disease stage was correlated with SDMT (R = -0.39, p = 0.036, pFDR = 0.079). In MS-DGM, disease stage was correlated with PASAT (R = -0.30, p = 0.018, pFDR = 0.045) and SDMT (R = -0.42, p = 0.027, pFDR = 0.065). No correlation was observed in MS-C.

The associations of disease stage in AQP4+ NMOSD without stratification by subtype were as follows. In AQP4+ NMOSD, disease stage was correlated with disease duration (R = 0.10, p = 0.047, pFDR = 0.14), number of relapses (R = 0.17, p = 0.0021, pFDR = 0.0085) and EDSS (R = 0.20, p < 0.0001, pFDR < 0.0001). In MS, disease stage was correlated with disease duration (R = 0.23, p < 0.0001, pFDR < 0.0001), number of relapses (R = 0.18, p = 0.0023, pFDR = 0.0041), EDSS (R = 0.15, p < 0.0001, pFDR < 0.0001), WMH volume (R = 0.22, p < 0.0001, pFDR < 0.0001), PASAT (R = -0.22, p < 0.0001, pFDR < 0.0001) and SDMT (R = -0.24, p = 0.012, pFDR = 0.020).


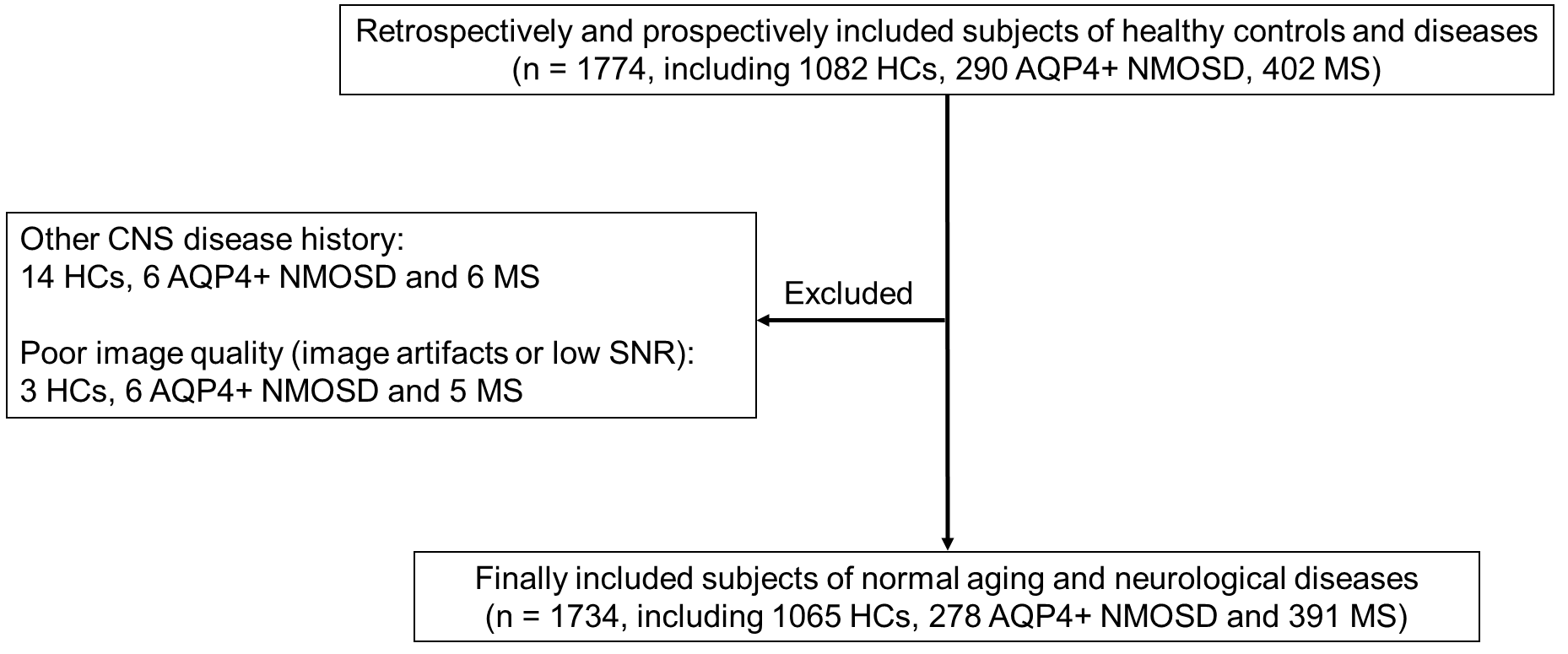


**Figure S1. A flowchart of the subjects included in this study.**


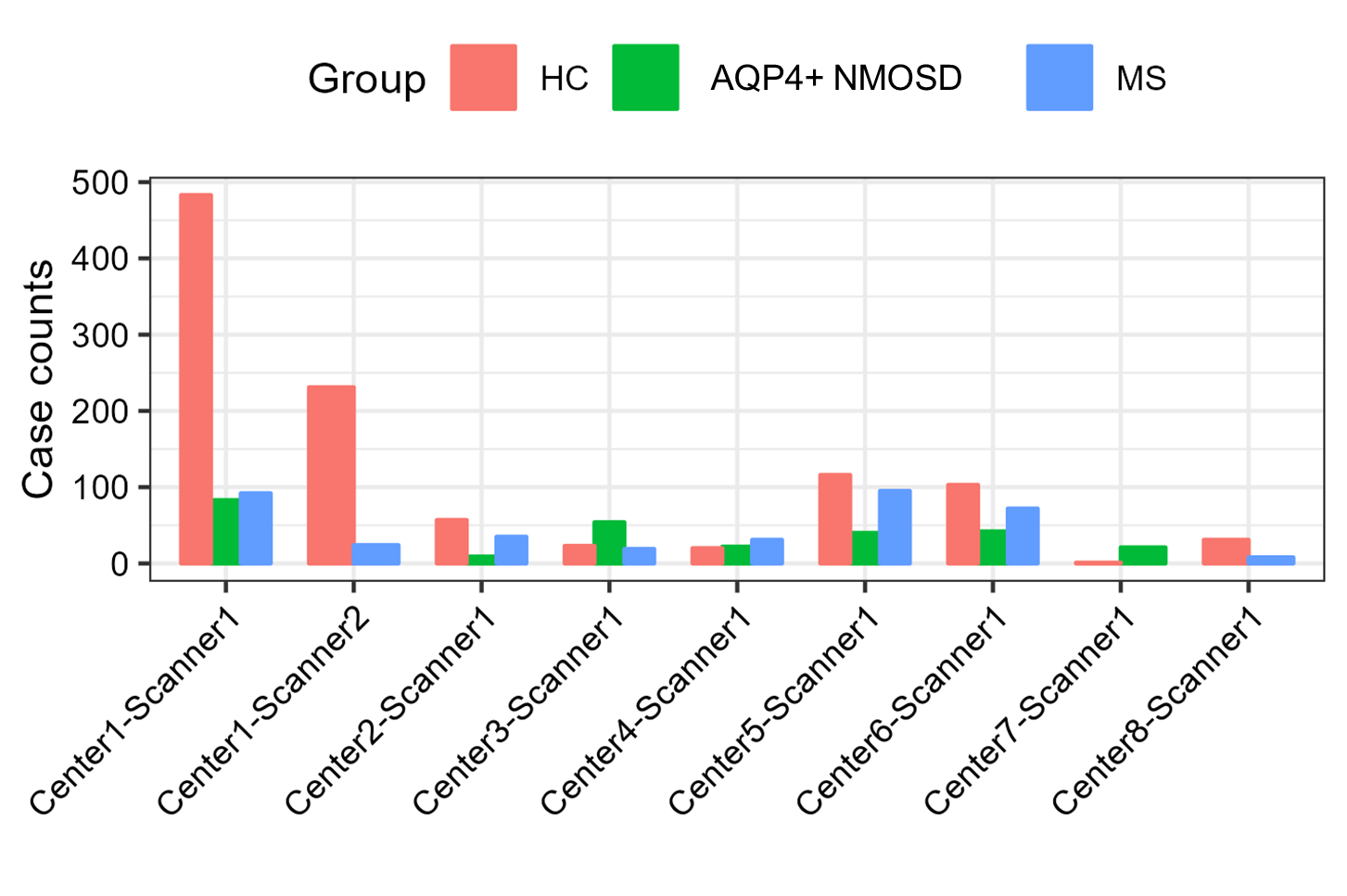


**Figure S2. The case distribution of the multicenter dataset.**


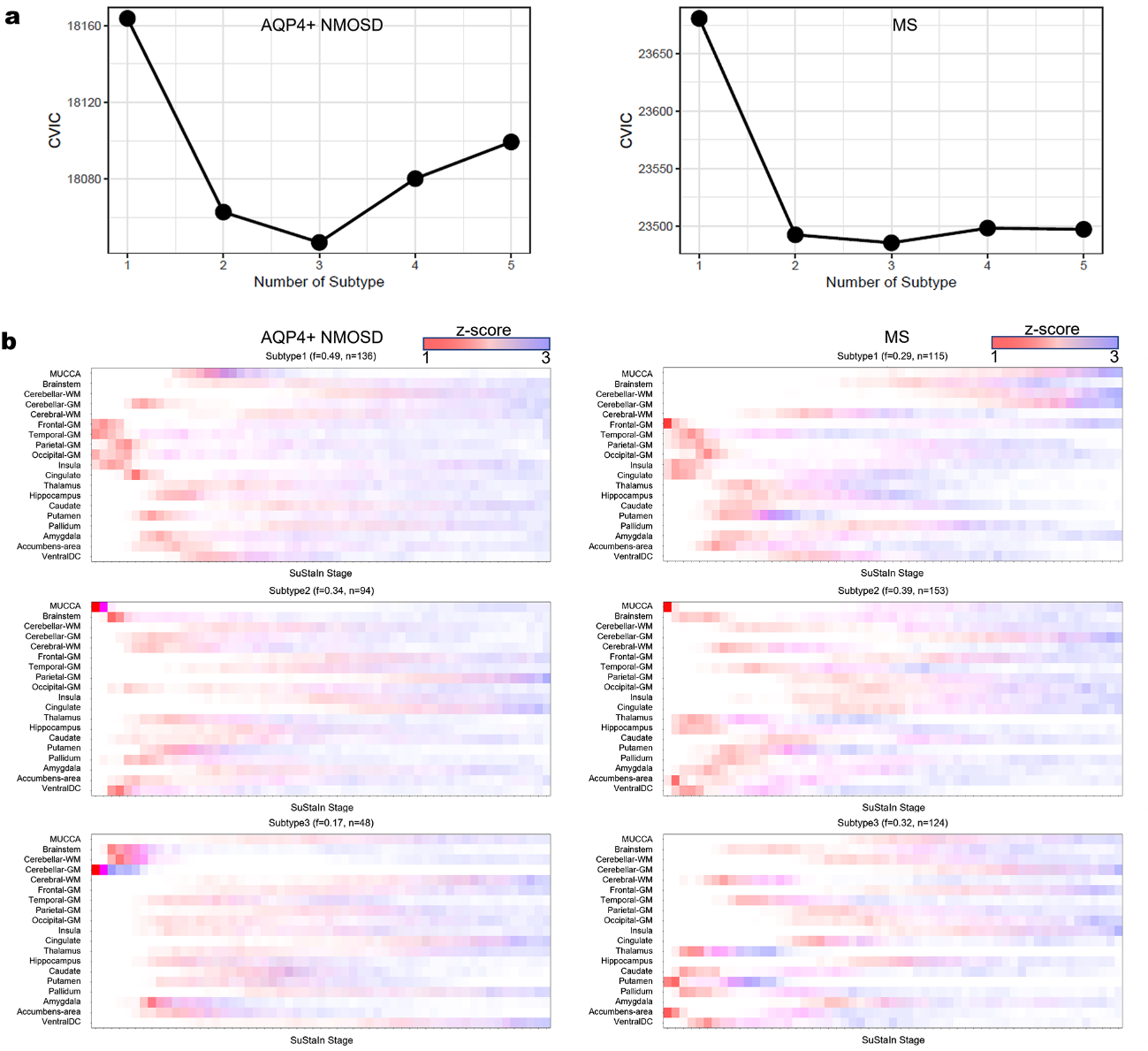


**Figure S3. Selection of the optimal number of subtypes by SuStaIn, and subtypes and stages of AQP4+ NMOSD and MS**. a, Cross-validation information criterion [CVIC]) to balance model complexity with internal model accuracy, and the subtype model was selected in a way to have the lowest CVIC; in cases with very similar CVIC, the model with lower complexity was adopted (e.g., fewer subtypes). b, Subtypes and stages of AQP4+ NMOSD and MS. AQP4+ NMOSD, aquaporin 4 antibody seropositive neuromyelitis optica spectrum disorders; MS, multiple sclerosis; MUCCA, mean upper cervical cord area; WM, white matter; GM, gray matter.


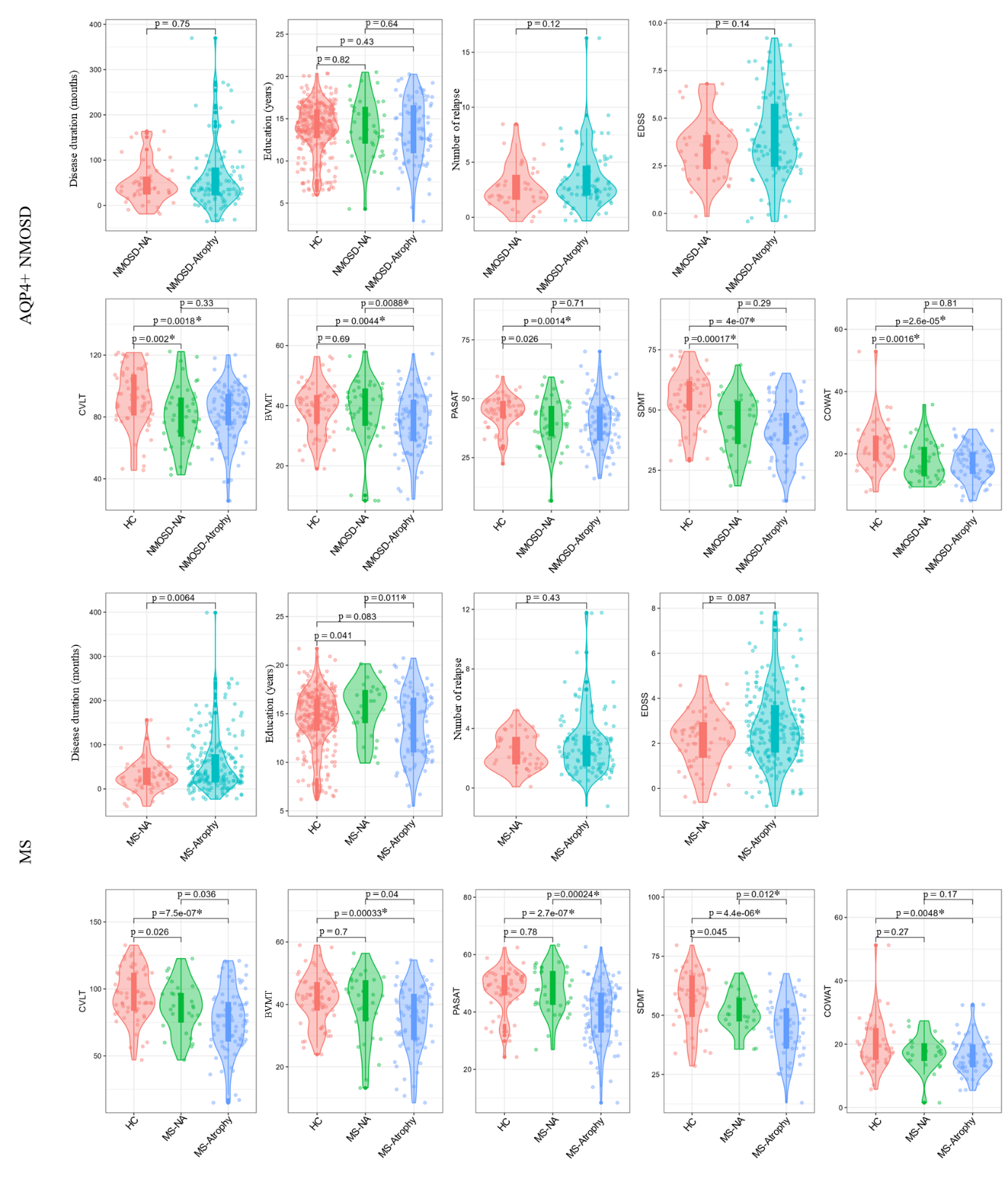


**Figure S4. The clinical and cognitive characteristics of the “normal appearing” AQP4+ NMOSD and MS patients compared to age- and sex- matched HCs and those with CNS atrophy.** HC, healthy control; AQP4+ NMOSD, aquaporin 4 antibody seropositive neuromyelitis optica spectrum disorders; MS, multiple sclerosis. * indicates statistical significance with pFDR < 0.05


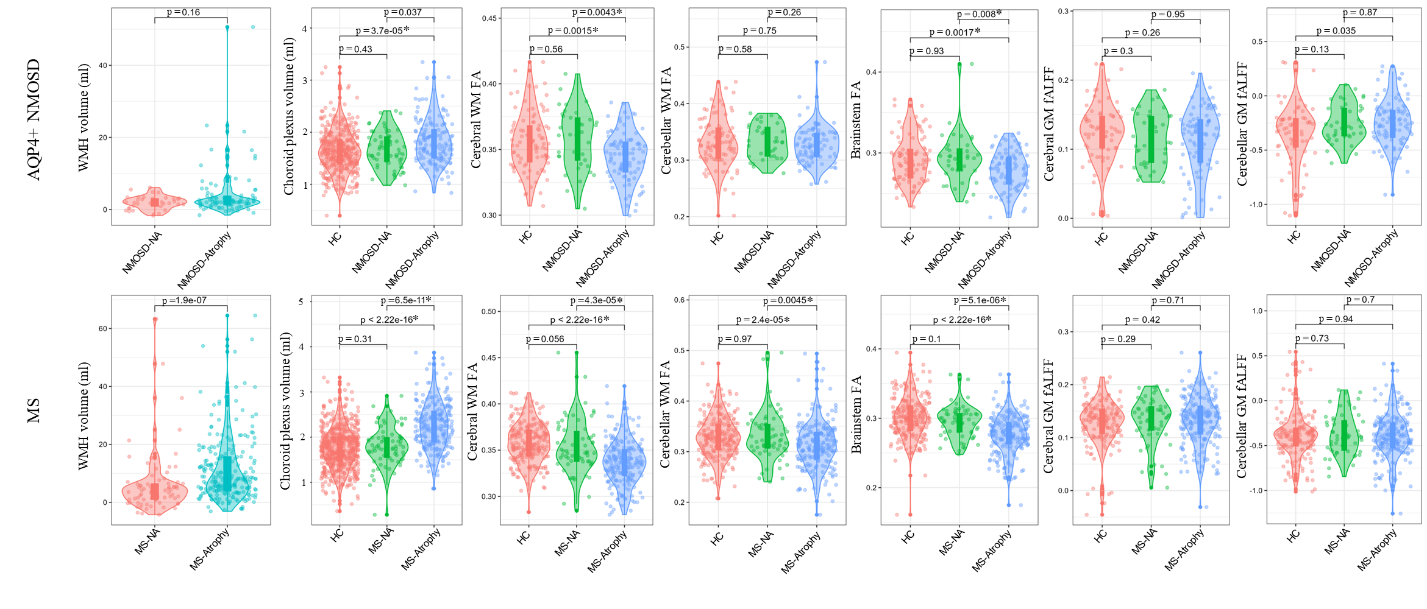


**Figure S5. The MR characteristics of the “normal appearing” AQP4+ NMOSD and MS patients compared to age- and sex- matched HCs and those with CNS atrophy.** HC, healthy control; AQP4+ NMOSD, aquaporin 4 antibody seropositive neuromyelitis optica spectrum disorders; MS, multiple sclerosis. * indicates statistical significance with pFDR < 0.05


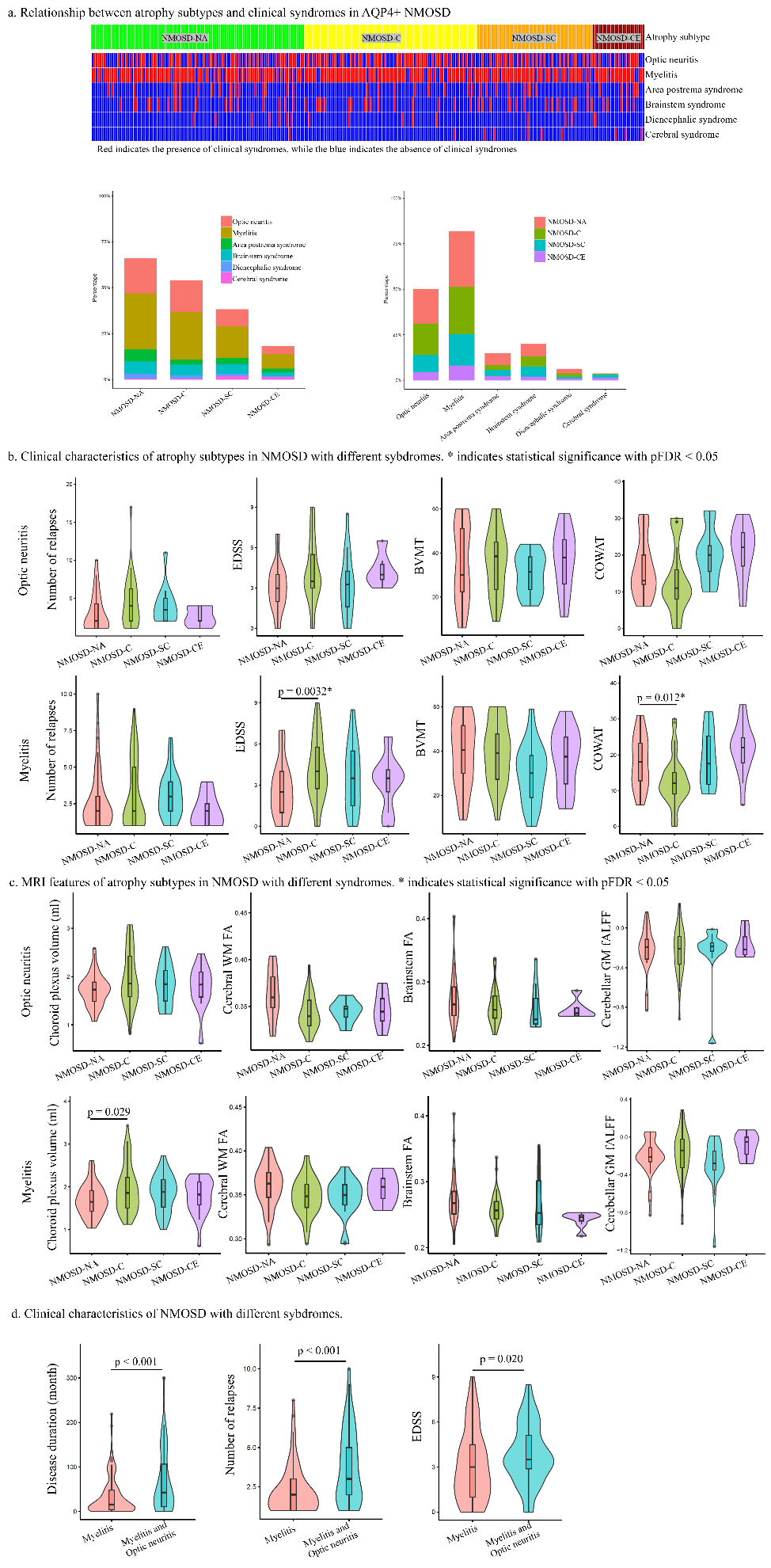


**Figure S6. The potential association analysis of atrophy subtypes and clinical syndrome in AQP4+ NMOSD.** AQP4+ NMOSD, aquaporin 4 antibody seropositive neuromyelitis optica spectrum; Brief BVMT, Visuospatial Memory Test-Revised; COWAT, Controlled Oral Word Association Test; EDSS, Expanded Disability Status Scale.

Table S1. Details of the MR protocols.

| Center Index | Scanner | 2D or 3D | FA (degree) | TR/TE (ms) | IR (ms) | Spatial Resolution (mm×mm×mm) | Matrix Size | Rs-fMRI dynamics | B values and directions |
| --- | --- | --- | --- | --- | --- | --- | --- | --- | --- |
| T1WI |  |  |  |  |  |  |  |  |  |
| 1 | Philips Ingenia CX | 3D | 8 | 7/3 | 880 | 1×1×1 | 256×256×196 |  |  |
| 1 | GE Premier | 3D | 12 | 7.3/3 | 450 | 1×1×1 | 256×256×176 |  |  |
| 2 | Siemens Skyra | 3D | 8 | 2300/2.3 | 900 | 1×1×1 | 256×256×192 |  |  |
| 3 | GE Discovery MR750 | 3D | 12 | 8.2/3.2 | 450 | 1×1×1 | 256×256×188 |  |  |
| 4 | Siemens TrioTim | 3D | 9 | 1600/2.1 | 1000 | 1×1×1 | 256×224×176 |  |  |
| 5 | GE Discovery MR750 | 3D | 12 | 8.2/3.2 | 450 | 1×1×1 | 256×256×196 |  |  |
| 6 | Siemens Skyra | 3D | 9 | 1900/2.3 | 900 | 1×1×1 | 256×256×176 |  |  |
| 7 | GE Discovery MR750 | 3D | 12 | 8.3/3.3 | 400 | 0.5×0.5×1 | 512×512×186 |  |  |
| 8 | Siemens Skyra | 3D | 9 | 1900/2.3 | 900 | 1×1×1 | 256×256×176 |  |  |
| FLAIR |  |  |  |  |  |  |  |  |  |
| 1 | Philips Ingenia CX | 3D | 90 | 4800/340 | 1650 | 0.6×0.6×1 | 400×400×165 |  |  |
| 1 | GE Premier | 3D | 90 | 5000/106 | 1522 | 1×1×1 | 256×256×170 |  |  |
| 2 | Siemens Skyra | 2D | 150 | 8000/84 | 2370 | 0.7×0.7×4 | 270×320×32 |  |  |
| 3 | GE Discovery MR750 | 2D | 142 | 8400/150 | 2100 | 0.5×0.5×4 | 512×512×32 |  |  |
| 4 | Siemens TrioTim | 2D | 120 | 7500/87 | 2500 | 0.5×0.5×4 | 512×512×35 |  |  |
| 5 | GE Discovery MR750 | 2D | 111 | 8800/150 | 2100 | 0.5×0.5×4 | 512×512×32 |  |  |
| 6 | Siemens Skyra | 2D | 150 | 9000/81 | 2500 | 0.7×0.7×4 | 320×320×35 |  |  |
| 7 | GE Discovery MR750 | 2D | 90 | 8000/130 | 2250 | 0.5×0.5×4 | 512×512×34 |  |  |
| 8 | Siemens Skyra | 2D | 120 | 7000/77 | 2500 | 0.4×0.4×2.6 | 512×408×45 |  |  |
| DTI |  |  |  |  |  |  |  |  |  |
| 1 | Philips Ingenia CX | 2D | 90 | 4000/88 |  | 2.5×2.5×2.5 | 96×96×60 |  | 1000, 48 |
| 2 | Siemens Skyra | 2D | 90 | 3700/95 |  | 1.7×1.7×5 | 128×128×25 |  | 1000, 62 |
| 3 | GE Discovery MR750 | 2D | 90 | 5800/70 |  | 2×2×3 | 128×128×48 |  | 1000, 50 |
| 4 | Siemens TrioTim | 2D | 90 | 11000/98 |  | 2×2×2 | 128×116×60 |  | 1000, 61 |
| 5 | GE Discovery MR750 | 2D | 90 | 4700/98 |  | 1×1×4 | 256×256×35 |  | 1000, 50 |
| 6 | Siemens Skyra | 2D | 90 | 8000/100 |  | 2×2×2.5 | 128×128×53 |  | 1000, 60 |
| 7 | GE Discovery MR750 | 2D | 90 | 15000/86 |  | 1×1×2.5 | 256×256×53 |  | 1000, 30 |
| Rs-fMRI |  |  |  |  |  |  |  |  |  |
| 1 | Philips Ingenia CX | 2D | 78 | 2000/30 |  | 3×3×4 | 80×80×40 | 180 |  |
| 2 | Siemens Skyra | 2D | 90 | 2500/30 |  | 3×3×3 | 70×70×43 | 200 |  |
| 3 | GE Discovery MR750 | 2D | 90 | 2000/45 |  | 3.5×3.5×4.5 | 64×64×32 | 180 |  |
| 4 | Siemens TrioTim | 2D | 90 | 2000/30 |  | 3.5×3.5×4 | 64×64×32 | 180 |  |
| 5 | GE Discovery MR750 | 2D | 90 | 2000/30 |  | 3.75×3.75×4 | 64×64×35 | 210 |  |
| 6 | Siemens Skyra | 2D | 90 | 2000/22 |  | 3.5×3.5×4 | 64×64×33 | 240 |  |
| 7 | GE Discovery MR750 | 2D | 90 | 2000/30 |  | 3.75×3.75×4 | 64×64×33 | 240 |  |

Note: DTI and rs-fMRI acquisition protocols were unavailable for center-1 GE Premier and Center-8 Siemens Skyra. T1WI, T1 weighted imaging; FLAIR, fluid attenuated inversion recovery; DTI, diffusion tensor imaging; rs-fMRI, resting state functional MRI; FA, flip angle; TR, time of repetition; TE, time of echo; IR, inversion recovery.
